# An Improved Multi-Strategy Crayfish Optimization Algorithm for Solving Numerical Optimization Problems

**DOI:** 10.3390/biomimetics9060361

**Published:** 2024-06-14

**Authors:** Ruitong Wang, Shuishan Zhang, Guangyu Zou

**Affiliations:** 1 Leicester Institution, Dalian University of Technology, Dalian 124221, China; 1160827774@mail.dlut.edu.cn (R.W.); coca820916@mail.dlut.edu.cn (S.Z.); 2 Institute of Public Foundations, Dalian University of Technology, Dalian 124221, China

**Keywords:** crayfish optimization algorithm, metaheuristic algorithm, cave candidacy strategy, fitness–distance balanced competition strategy, food covariance learning strategy, optimal non-monopoly search strategy

## Abstract

The crayfish optimization algorithm (COA), proposed in 2023, is a metaheuristic optimization algorithm that is based on crayfish’s summer escape behavior, competitive behavior, and foraging behavior. COA has a good optimization performance, but it still suffers from the problems of slow convergence speed and sensitivity to the local optimum. To solve these problems, an improved multi-strategy crayfish optimization algorithm for solving numerical optimization problems, called IMCOA, is proposed to address the shortcomings of the original crayfish optimization algorithm for each behavioral strategy. Aiming at the imbalance between local exploitation and global exploration in the summer heat avoidance and competition phases, this paper proposes a cave candidacy strategy and a fitness–distance balanced competition strategy, respectively, so that these two behaviors can better coordinate the global and local optimization capabilities and escape from falling into the local optimum prematurely. The directly foraging formula is modified during the foraging phase. The food covariance learning strategy is utilized to enhance the population diversity and improve the convergence accuracy and convergence speed. Finally, the introduction of an optimal non-monopoly search strategy to perturb the optimal solution for updates improves the algorithm’s ability to obtain a global best solution. We evaluated the effectiveness of IMCOA using the CEC2017 and CEC2022 test suites and compared it with eight algorithms. Experiments were conducted using different dimensions of CEC2017 and CEC2022 by performing numerical analyses, convergence analyses, stability analyses, Wilcoxon rank–sum tests and Friedman tests. Experiments on the CEC2017 and CEC2022 test suites show that IMCOA can strike a good balance between exploration and exploitation and outperforms the traditional COA and other optimization algorithms in terms of its convergence speed, optimization accuracy, and ability to avoid premature convergence. Statistical analysis shows that there is a significant difference between the performance of the IMCOA algorithm and other algorithms. Additionally, three engineering design optimization problems confirm the practicality of IMCOA and its potential to solve real-world problems.

## 1. Introduction

The optimization problem forms a specialized class of mathematical problems aimed at improving a system or solution by finding the best solution among multiple alternatives where the intended design objective is maximized or minimized. Frequently, real-world applications require the imposition of multiple design constraints in order to satisfy the solution’s usability requirements. Most of these problems are nonlinear, complex, and non-microscopic, making them challenging to solve. To solve such problems, two main types of algorithms are available. One type is deterministic algorithms. A major drawback of these methods is that they rely on a priori information about the derivatives of the functions involved in the problem, including the objective function and constraints. In many cases, these traditional optimization methods have difficulty in determining the optimal solution because they terminate the search process when the gradient approaches zero, which occurs in both global and local optimal cases, resulting in their very low efficiency. The other category is metaheuristics, which are general-purpose algorithms developed for solving highly complex optimization problems by manipulating one or more potential solutions to achieve the optimal or most advantageous solution. Their iterative approach aims to progress from an initial solution of poor quality to an optimal solution, ending the process once some predetermined criterion has been reached. These algorithms have proven their reliability and effectiveness in discovering optimal solutions to complex real-world problems. In addition to classical numerical optimization, metaheuristic algorithms have demonstrated their efficacy in a wide range of optimization tasks, including, but not limited to, feature selection [1,2], the traveling salesman problem [3,4], image segmentation problems [5,6,7], wireless sensor coverage problems [8,9], parameter estimation for solar photovoltaic models [10,11], and path planning [12,13]. In the past decades, new metaheuristic algorithms have been continuously proposed that are based on evolutionary theory, physical laws, biological population behavior, and human behavior. Table 1 shows some of the algorithms proposed in recent years.

Metaheuristics are randomized algorithms, and that randomness often leads to better solutions. They involve two important search phases: exploration and exploitation. The exploration phase requires the algorithm to explore various regions of the search space to discover each promising region, while the exploitation phase involves continuing the search within the potential regions discovered in the exploration phase to find the best solution. According to the No Free Lunch (NFL) theorem [37], no class of algorithms can solve all problems well. The theory reflects the expandability of algorithms. Therefore, scholars continue to propose various novel or improved optimization algorithms to solve different optimization problems.

The crayfish optimization algorithm (COA) [38] is a swarm-based metaheuristic algorithm proposed by Jia et al. The algorithm is modeled by simulating the thermal, competitive, and foraging behaviors of crayfish in summer. As a new swarm-based metaheuristic algorithm, COA suffers from the drawbacks such as slow convergence, low accuracy, and tendency to fall into the local optimum when dealing with real complex problems. In order to overcome these shortcomings, an improved multi-strategy crayfish optimization algorithm (IMCOA) is proposed in this paper. First, to address the imbalance between local exploitation and global exploration in the summer heat avoidance phase and the competition phase, a cave candidate strategy and a fitness–distance balanced competition strategy are proposed, respectively, so that these two behaviors can better coordinate the global and local optimization capabilities and avoid falling into the local optimum prematurely. Second, a food covariance learning strategy is introduced in the foraging phase to enhance the population diversity and to improve the convergence accuracy and convergence speed. Finally, an optimal non-monopoly search strategy is introduced to perturb the updated optimal solution, which improves the ability of the algorithm to obtain the global optimal solution.

In the experimental part, the optimization performance of IMCOA is examined using CEC2017 and CEC2022 test functions. The superiority of the method in this paper is illustrated by numerical analyses, convergence analyses, stability analyses, Friedman tests and Wilcoxon rank–sum tests.

The main contributions of this paper are as follows.

1. A cave candidate strategy and fitness–distance balanced competition strategy are proposed to adjust the original COA heat avoidance behavior and competition behavior to improve the optimization ability of the algorithm and effectively prevent the risk of the algorithm falling into a local optimum.
2. The direct foraging behavior formula of COA is optimized using the food covariance learning strategy to improve the quality of understanding and search efficiency.
3. An optimal non-monopoly search strategy is introduced to perturb the updated optimal solution, which improves the ability of the algorithm to obtain the global optimal solution and enables the algorithm to explore more valuable domains.
4. IMCOA is tested on 29 functions of CEC2017 and 12 functions of CEC2022 with different dimensions.
5. IMCOA is tested on three engineering applications.

The rest of the paper is specifically structured as follows: Section 2 describes the basics of the COA algorithm. Section 3 presents the details of the proposed improved strategy followed by a time complexity analysis. Section 4 and Section 5 are the experimental parts, where the performance of the proposed IMCOA is evaluated by the CEC2017 and CEC2022 test suites and compared with other algorithms. Additionally, three engineering design optimization problems are employed to evaluate the performance of IMCOA. We summarize our study in Section 5 and present an outlook for future research.

## 2. Crayfish Optimization Algorithm (COA)

This section describes the mathematical model of COA in detail, including the processes of the initialization phase, the exploration phase, and the exploitation phase.

### 2.1. Initialization Phase

COA is a swarm-based optimization algorithm that utilizes randomly initialized crayfish groups as search agents. This is represented by Equation (1):

(1) $$X=lb+rand\times \left(ub-lb\right)$$

In this equation, rand represents a random number in [0, 1]. ub and lb denote the upper and lower bounds of the solution space, respectively. In each iteration, each *X* generates a candidate solution. The best solution obtained so far is considered the minimum solution.

The behavior of crayfish enters different stages with change in temperature. The temperature is calculated as shown in Equation (2). In COA, when the temperature is higher than 30 °C, crayfish will choose heat avoidance behavior. Otherwise, crayfish will perform foraging behavior. Crayfish show strong foraging behavior between 20 °C and 30 °C. The optimal feeding temperature is 25 °C. Therefore, COA defines the temperature range as 20 °C to 35 °C. The mathematical model of crayfish feeding is shown in Equation (3).

(2) temp=rand×15+20

(3) $$prop=C_{1}\times \left(\frac{1}{\sqrt{2\pi }}\times \exp\left(-\frac{{\left(temp-25\right)}^{2}}{2\sigma^{2}}\right)\right)$$

where σ and $C_{1}$ are two constant values used to control crayfish feeding at different temperatures.

### 2.2. Heat Avoidance Behavior

During the heat avoidance behavior phase, when the temperature is greater than 30 degrees, crayfish recognize that the temperature is too high. At this time, crayfish will choose a relatively cool place to avoid the heat.

The mathematical model of crayfish summering behavior is shown in Equation (4):

(4) $$X_{cave}=\frac{\left(X_{Gbest}+X_{Lbest}\right)}{2}$$

where $X_{Gbest}$ denotes the best position obtained by the number of iterations so far, and $X_{Lbest}$ represents the best position of the current crayfish population.

During the summer vacation phase, two cases occur. One is when rand<0.5, i.e., there are no other crayfish in the cave; the crayfish directly enter the cave to avoid the heat, and the mathematical model is shown in Equation (5). The other is when rand≥0.5. When this occurs, crayfish compete with each other. The representation of this competitive behavior is shown in Equation (6).

(5) $$X_{new}=X_{i,j}+\left(2-\left(\frac{t}{T}\right)\right)\times rand\times \left(X_{cave}-X_{i,j}\right)$$

(6) $$X_{new}=X_{i,j}-X_{z,j}+X_{cave}$$

where t denotes the current number of iterations and T denotes the maximum number of iterations. $X_{z,j}$ is a randomly selected agent from the crayfish.

### 2.3. Foraging Behavior

When the temperature is less than or equal to 30 °C, the temperature is suitable for crayfish to forage. The crayfish will move toward the food. After finding the food, the crayfish will judge the size of the food by Equation (7); if the food is too big, the crayfish will tear the food with its claws and then eat the food with the second claw and the third claw alternately.

(7) $$Q=C_{2}\times rand\times \left(\frac{F\left(X\right)}{F\left(X_{Gbest}\right)}\right)$$

where $C_{2}$ is the food factor representing the largest food, which has a constant value of 3; $F\left(X\right)$ is the fitness value of the crayfish; and $F\left(X_{Gbest}\right)$ is the fitness value of the global best position.

When $Q>\left(C_{2}+1\right)/2$, the crayfish tears up the food and then eats alternately with the second claw and the third claw. The foraging equation at this stage is expressed as follows:

(8) $$X_{new}=X_{i,j}+\exp\left(-\frac{1}{Q}\right)\times X_{Gbest}\times prop\times \left(\cos\left(2\times \pi \times rand\right)-\left(\sin2\times \pi \times rand\right)\right)$$

When $Q\leq \left(C_{2}+1\right)/2$, the crayfish only needs to move toward the food and eat directly, and Equation (9) is as follows:

(9) $$X_{new}=\left(X_{i,j}-X_{Gbest}\right)\times prop+prop\times rand\times X_{i,j}$$

The pseudo-code of the COA is shown in Algorithm 1:
**Algorithm 1** Crayfish Optimization Algorithm (COA)Initialization: Iterations *T*, Population *Np*, Dimension *Dim*; Randomly generate an initial population and assess the fitness values to obtain *X_Gbest_* and *X_Lbest_*While (*t* < *T*) do  Defining temperature *temp* by Equation (2)  If *temp* > 30    Define a cave *X*_cave_ based on Equation (4)    If *rand* < 0.5     Update the crayfish position according to Equation (5)    Else     Update the crayfish position according to Equation (6)   End  Else   Calculate food intake *prop* and food size *Q* are obtained by Equations (3) and (7)   If *Q* > (*C*_3_ + 1)/2    Update the crayfish position according to Equation (8)   Else    Update the crayfish position according to Equation (9)   End  End  Update fitness values, *X_Gbest_*, *X_Lbest_*  *t* = *t* + 1End while

## 3. The Proposed IMCOA

This section details the four improvement strategies proposed in this paper, incorporating the cave candidacy strategy, fitness–distance balanced competition strategy, food covariance learning strategy, and optimal non-monopoly search strategy. The time complexity of the IMCOA algorithm proposed in this paper as well as the original COA algorithm is also analyzed to ensure that the algorithm proposed in this paper improves the performance of the original algorithm without significantly increasing the time complexity, which is within the acceptable range.

### 3.1. Cave Candidacy Strategy

In COA, the summering phase of the formulation introduces the optimal solution, which facilitates the enhancement of COA exploitation. However, if the optimal solution falls into a local optimum, it will cause the remaining agents to follow to perform the optimal trap. In addition, the overpowering exploitation capability of the exploration phase is not conducive to balancing the search in this phase. In order to ensure that the exploration phase has a certain amount of exploitation capability along with a good exploration capability, we propose a cave candidate strategy. These candidate caves consist of several different categories of caves, and the crayfish can choose one from the candidate caves to enter. The specific formula of this strategy is expressed as follows:

(10) $$X_{cc}=Select{\left[X_{Gbest},X_{2},X_{3},X_{4},X_{5},X_{6},X_{cave}\right]}_{random}$$

(11) $$X_{4}=\left(X_{Gbest}+X_{2}\right)/2$$

(12) $$X_{5}=\left(X_{Gbest}+X_{2}+X_{3}\right)/3$$

(13) $$X_{6}=\sum_{i=1}^{0.35Np}\omega_{i}\times X_{i}$$

(14) $$\omega_{i}=\frac{\ln(0.35Np+0.5)-\ln(i)}{\sum_{i=1}^{NP/2}(\ln(0.35Np+0.5)-\ln(i))}$$

where $X_{2}$ and $X_{3}$ are the second and third global best agents. $X_{4}$ and $X_{5}$ are composed similarly to $X_{cave}$ and are used to enhance the exploitation capability. $X_{6}$ is obtained from a weighted average of the best part of the agents in the crayfish according to their performance, which can represent the evolutionary trend of the crayfish. By introducing the cave candidacy strategy, it makes the cave selection of crayfish more diverse and ensures the performance of the algorithm. The modified heat avoidance formula is expressed as follows:

(15) $$X_{new}=X_{i,j}+\left(2-\left(\frac{t}{T}\right)\right)\times rand\times \left(X_{cc}-X_{i,j}\right)$$

### 3.2. Fitness–Distance Balanced Competition Strategy

The updating method of the competition phase is mainly used to further search the problem space, but the original formula places too much emphasizes on random searching, which weakens the exploitation. Therefore, a fitness–distance balanced competition strategy is used in this paper, which selects an agent based on both fitness and distance scores together. In order to ensure sufficient exploitation performance in the competition phase, along with some global exploration performance, the agent is further filtered using a roulette wheel to ensure that every agent has a chance to be selected. This properly synchronizes the exploitation and exploration performance. The modified competition stage formula is shown in Equation (17).

(16) $$X\left(Score\right)=\left(norm\left(Fit\right)+norm\left(Dis\right)\right)/2$$

(17) $$X_{Fdbc}=Select{\left[X\right]}_{roulette}$$

(18) $$X_{new}=X_{i,j}-X_{Fdbc,j}+X_{cave}$$

where $norm\left(Fit\right)$ is the normalized value of fitness and $norm\left(Dis\right)$ is the normalized value of distance. $X_{Fdbc}$ is the agent selected using the strategy.

### 3.3. Food Covariance Learning Strategy

In COA, the foraging phase is categorized by two methods. By analyzing the distribution of PROP, it is found that COA executes Equation (9) more often. This approach accelerates the convergence, but it is easy to fall into the local optimum, which is not favorable for further exploitation. Based on the above analysis, this paper proposes a food covariance learning strategy. This strategy will utilize the effective information of the better part of the agents besides utilizing the information of the food source (optimal solution) [39]. This can correctly guide the agent evolution and improve the algorithm performance.

(19) $$Xnew=\left(X_{Gbest}+X_{mean}+X\right)/3+y,y\sim N\left(0,Cov\right)$$

(20) $$Cov(i)=\frac{1}{0.35Np}\sum_{i=1}^{0.35Np}\left(X-X_{mean}\right)\times {\left(X-X_{mean}\right)}^{T}$$

(21) $$X_{mean}=\sum_{i=1}^{0.35Np}\omega_{i}\times X_{i}$$

This strategy mainly utilizes the overall distribution information of the better agents, which is detrimental to the performance of the strategy when the overall number is small. Therefore, this paper further introduces an external archiving mechanism, which puts the best part of agents in each iteration into the archive. When the number of archived agents is large, the redundant agents are removed according to the first-in-first-out principle.

### 3.4. Optimal Non-Monopoly Search Strategy

The quality of the optimal solution has an important impact on the performance of the algorithm, and if the optimal solution falls into a local optimum, it will cause the subsequent agents to fall into a local optimum as well. In order to avoid premature convergence and to improve the global search capability, the optimal solution needs to be handled. The non-monopoly search strategy [40] is a new local search method that modifies each dimension of the current solution space along the search space, which can further improve the quality of the optimal solution. In this paper, we utilize the non-monopoly search strategy to perturb the optimal individuals to further enhance the performance. The non-monopoly search strategy is formulated as follows:

(22) $$X_{Gnew}(j)=rand\times X_{Gbest}(random)$$

(23) $$X_{Gnew}(j)=X_{Gbest}(j)-\left(X_{Gbest}(RS)\times rand\right)\times eps-\left(X_{Gbest}(j)-1\right)$$

where $X_{Gnew}(j)$ is the j dimension of the new solution. $X_{Gbest}(random)$ is a random dimension of the best solution. In this strategy, Equation (22) is used in pre-period, and Equation (23) is used in post-period. In order to further enhance the global search capability in the period and the local search capability in the post period, in this paper, the Cauchy operator and the Gaussian operator are introduced. The Cauchy operator can effectively provide the agent with a wide range of perturbations, while the Gaussian operator can provide the agent with finer adjustments. The modified mathematical formulas are represented as follows:

(24) $$X_{Gnew}(j)=Cauchy\times X_{Gbest}(random)$$

(25) $$X_{Gnew}(j)=X_{Gbest}(j)-\left(X_{Gbest}(RS)\times rand\right)\times eps-\left(X_{Gbest}(j)-1\right)\times Gaussian$$

### 3.5. Implementation Steps of the IMCOA Algorithm

**Figure 1:**
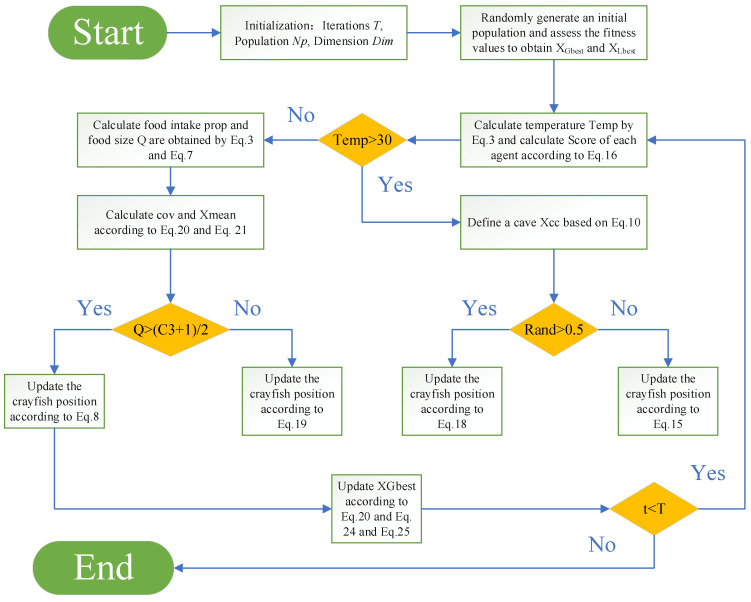
The flow chart of IMCOA.

The pseudo-code and flow chart of the IMCOA is shown in Algorithm 2 and Figure 1.
**Algorithm 2** Improved Multi-strategy Crayfish Optimization Algorithm (IMCOA)Initialization: Iterations *T*, Population *Np*, Dimension *Dim*; Randomly generate an initial population and assess the fitness values to obtain *X_Gbest_* and *X_Lbest_*While (*t* < *T*) do  Defining temperature *temp* by Equation (2)  Calculate Score of each agent according to Equation (16)  If *temp* > 30    Define a cave *X*_cc_ based on Equation (10)    If *rand* < 0.5     Update the crayfish position according to Equation (15)    Else     Update the crayfish position according to Equation (18)   End  Else   Calculate food intake *prop* and food size *Q* are obtained by Equations (3) and (7)   Calculate cov and X_mean_ according to Equations (20) and (21)   If *Q* > (*C*_3_ + 1)/2     Update the crayfish position according to Equation (8)   Else     Update the crayfish position according to Equation (19)   End  End  Update fitness values, *X_Gbest_*, *X_Lbest_*  Update *X_Gbest_* according to Equations (20), (24) and (25)  *t* = *t* + 1End while

### 3.6. Computational Time Complexity

The time complexity reflects the processing length needed for an algorithm to resolve a problem when its scale is increasing. As for COA, the population is N, the dimension is D, and the maximum number of iterations is T. During the initialization, the time complexity for fitness calculation is $O\left(N\times D\right)$. The individual position update involves updating the positions of each individual over T iterations, so the time complexity is $O\left(T\times N\times D\right)$. Therefore, the total time complexity of COA is $O\left(N\times D+T\times N\times D\right)$. Removing lower-order terms, the overall time complexity of COA can be simplified as $O\left(T\times N\times D\right)$.

For IMCOA, the initialization process is $O\left(N\times D\right)$. The time complexity of the cave candidacy strategy and fitness–distance balanced competition strategy is $O\left(T\times N/2\times D\right)$, the food covariance learning strategy time complexity is $O\left(T\times N/2\times D^{2}\right)$, and the optimal non-monopoly search strategy time complexity is $O\left(T\times D^{2}\right)$. Thus, the total complexity of IMCOA is as below:

$$O\left(IMCOA\right)=O\left(N\times D+T\times N/2\times D+T\times N/2\times D^{2}+T\times D^{2}\right)=O\left(TD^{2}\times \left(N/2+1\right)\right)$$

Although the time complexity of IMCOA is larger than that of COA, the IMCOA performance is significantly enhanced compared with that of COA, so this case can be accepted.

## 4. Experimental Results and Discussion of the CEC2017 Test Suite

### 4.1. Parameter Setting and Environment

The software and hardware used for all experiments are given in Table 2. The experimental parameters of this paper set the population number to 50, the maximum number of iterations to 500, and each algorithm was set to run independently 30 times and to select and record the average value, best value, and standard deviation in each run. The parameters of each algorithm are set out in Table 3.

### 4.2. CEC2017 Benchmark Function Experiment

#### 4.2.1. Description of Benchmark Functions

The benchmark test function serves as a crucial tool for evaluating the performance of algorithms, offering a standardized platform to assess and compare various optimization optimizers. In this study, we utilize the CEC2017 test suite to evaluate the performance of the proposed IMCOA algorithm across dimensions of 30, 50, and 100. With increasing dimensionality, the number of local optimal solutions also increases, enabling the suite to effectively evaluate the algorithm’s global optimization capability. The basic content of the CEC2017 benchmark functions are shown in Table A1 in Appendix A.

#### 4.2.2. Case 1: CEC2017 (Dim = 30)

##### Quantitative Analysis

**Figure 2:**
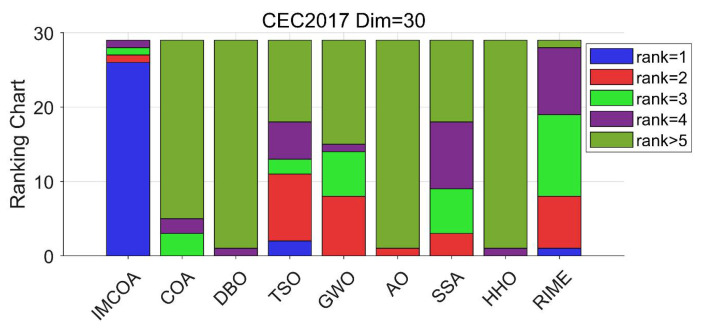
CEC2017 ranking stack chart (Dim = 30).

The results of this experimental case are contained in Table A2 in Appendix A. IMCOA achieved the best results for F1–F8, F10, F11, F13–F20, and F22–F29. TSO performed the best for F12 and F21. RIME provided the best solution for F9. The other algorithms did not achieve the best results for any function. In particular, it is noted that IMCOA outperformed COA on all functions, which indicates that the improvement strategy proposed in this paper effectively improves the performance of COA in 10 dimensions. To visualize the ranking of each algorithm, stacked bar charts were drawn based on the ranking of each algorithm, as shown in Figure 2. We categorized the rankings into five major categories: average rank first, average rank second, average rank third, average rank fourth, and other rankings. As can be seen in Figure 2, the IMCOA rankings are significantly higher compared with the COA rankings.

**Figure 3:**
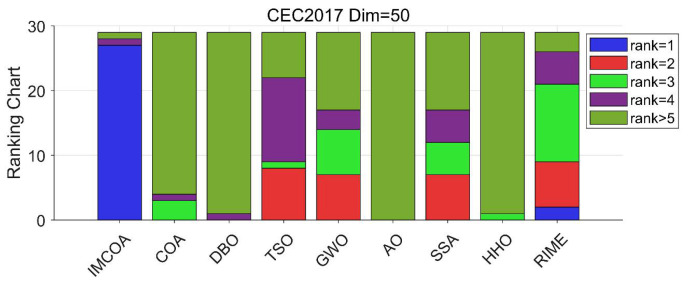
CEC2017 ranking stack chart (Dim = 50).

**Figure A1:**
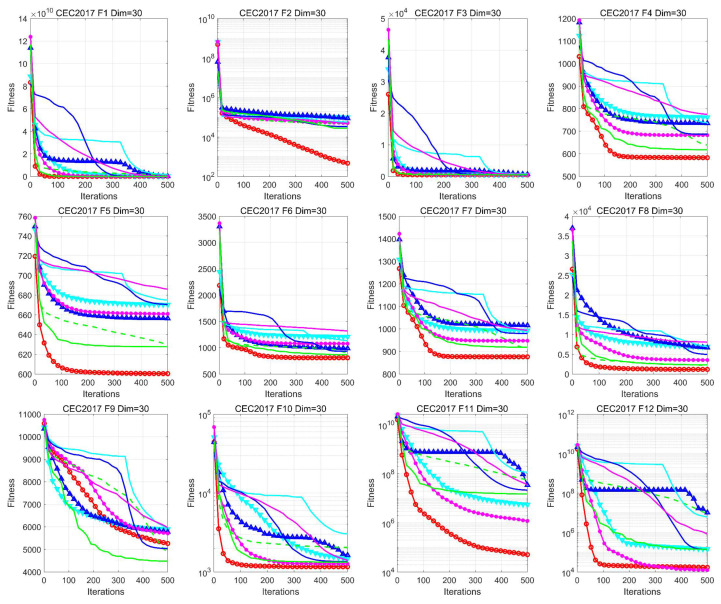

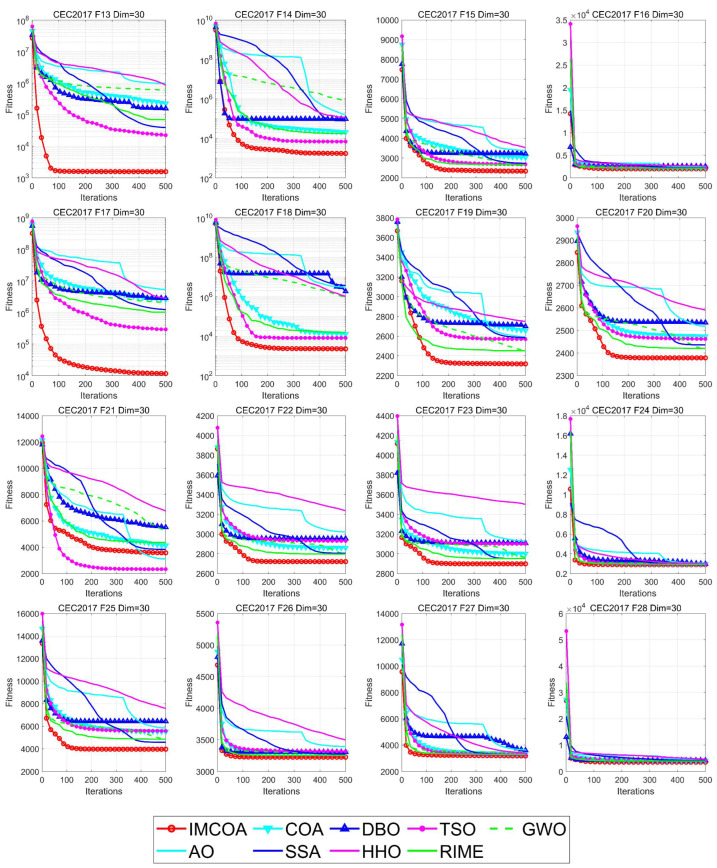
Comparison of convergence curves of the 9 algorithms using CEC2017 (Dim = 30).

Figure A1 in Appendix A shows the average fitness convergence curves for IMCOA and the comparison algorithm with Dim = 10. As can be seen in Figure 3, IMCOA can break away from the local optimum and find better solutions in most functions, and it has the fastest convergence rate. This shows the effectiveness of the four strategies proposed in this paper. These strategies not only free the algorithm from local optimality, but they also improve the convergence speed and accuracy of the algorithm.

**Figure A2:**
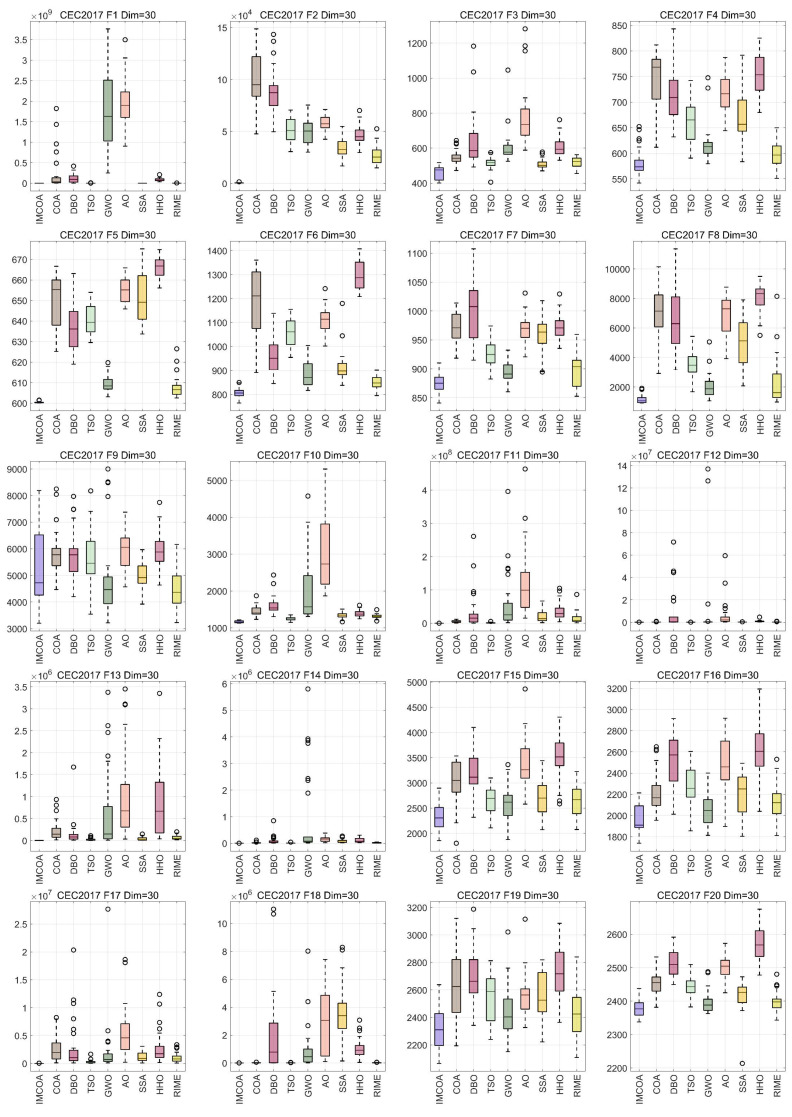

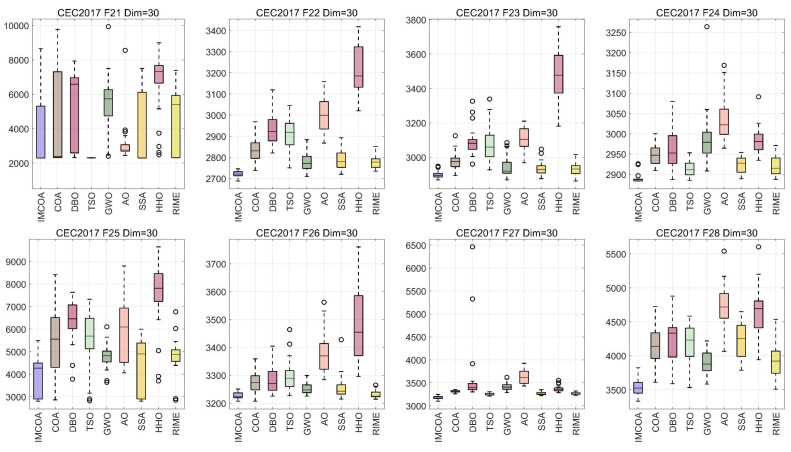
Comparison of box diagrams of the 9 algorithms using CEC2017(Dim = 30).

In Figure A2 in Appendix A, the performance of the nine algorithms is presented in detail in box-and-line plots, and it is clear that IMCOA has the best performance. The distribution of solutions for IMCOA is more centralized and smaller than that of all the other algorithms, which demonstrates the excellent performance of the IMCOA algorithm in terms of global exploration and local exploitation and verifies its effectiveness and accuracy.

##### Statistical Analysis

In this section, we will utilize the Wilcoxon rank–sum test and Friedman test to analyze the experimental results, assessing the statistical differences between IMCOA and other comparative algorithms.

In the rank–sum test, the significance level is defined as 5%. If the calculated value is less than 5%, this indicates that there is a significant difference between the comparison algorithms. If it is greater than 5%, it proves that there is no significant difference between the two algorithms. The symbols “+/=/−” are used to indicate whether IMCOA’s performance is superior, equivalent, or inferior to its competitors. As can be seen in Table 4 and Table 5, the similarity between IMCOA and the seven algorithms being compared is low, mostly below 5%. This indicates that there are differences between the optimization results of IMCOA and the other algorithms. The results of the Friedman test for each algorithm are also recorded in Table 5. IMCOA is ranked first in 30 dimensions and COA is ranked sixth.

#### 4.2.3. Case 2: CEC2017 (Dim = 50)

##### Quantitative Analysis

The results of this experimental case are shown in Table A3 in Appendix A. IMCOA achieved the best results for all functions except F9 and F21. RIME showed the best performance for F9 and F21. The rest of the algorithms did not rank first for any function. Compared with COA, IMCOA obtained a big boost in ranking. Similarly, it can be noticed that whenever IMCOA achieved the best results, it achieved the best results in all metrics for all the functions. Figure 3 plots a bar chart based on the rankings of the different algorithms. It can be seen that IMCOA has a significant advantage.

**Figure A3:**
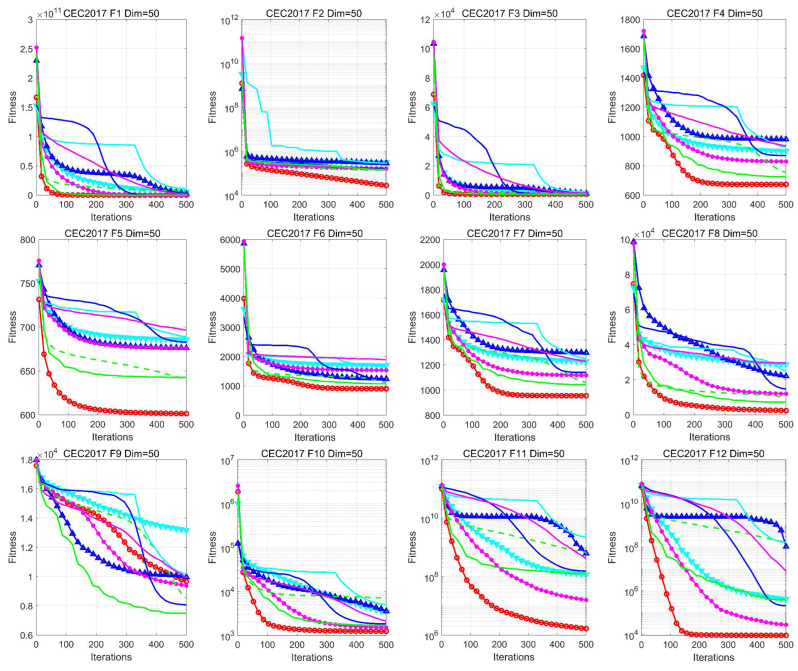

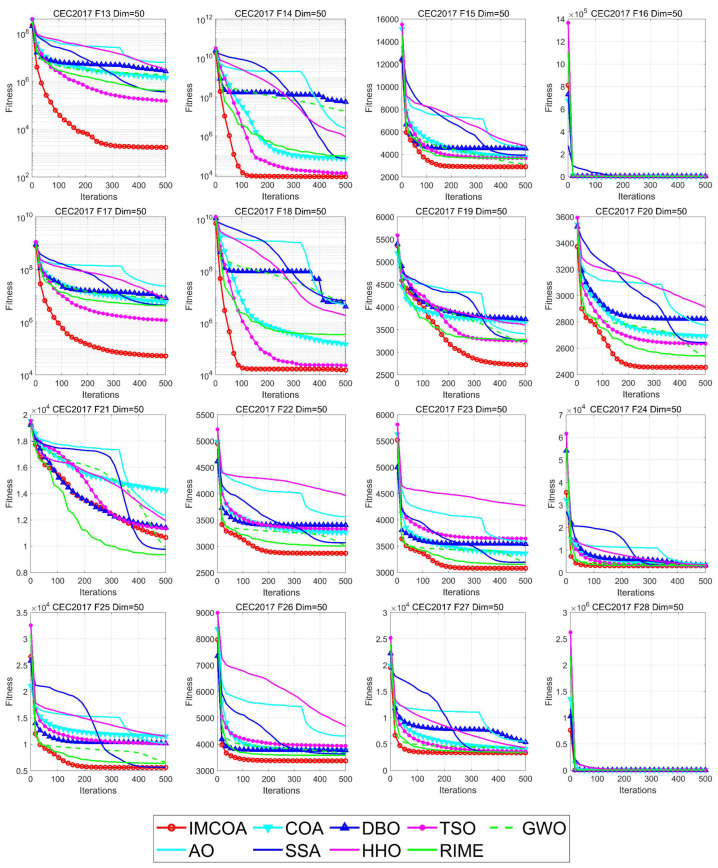
Comparison of convergence curves of the 9 algorithms using CEC2017(Dim = 50).

**Figure A4:**
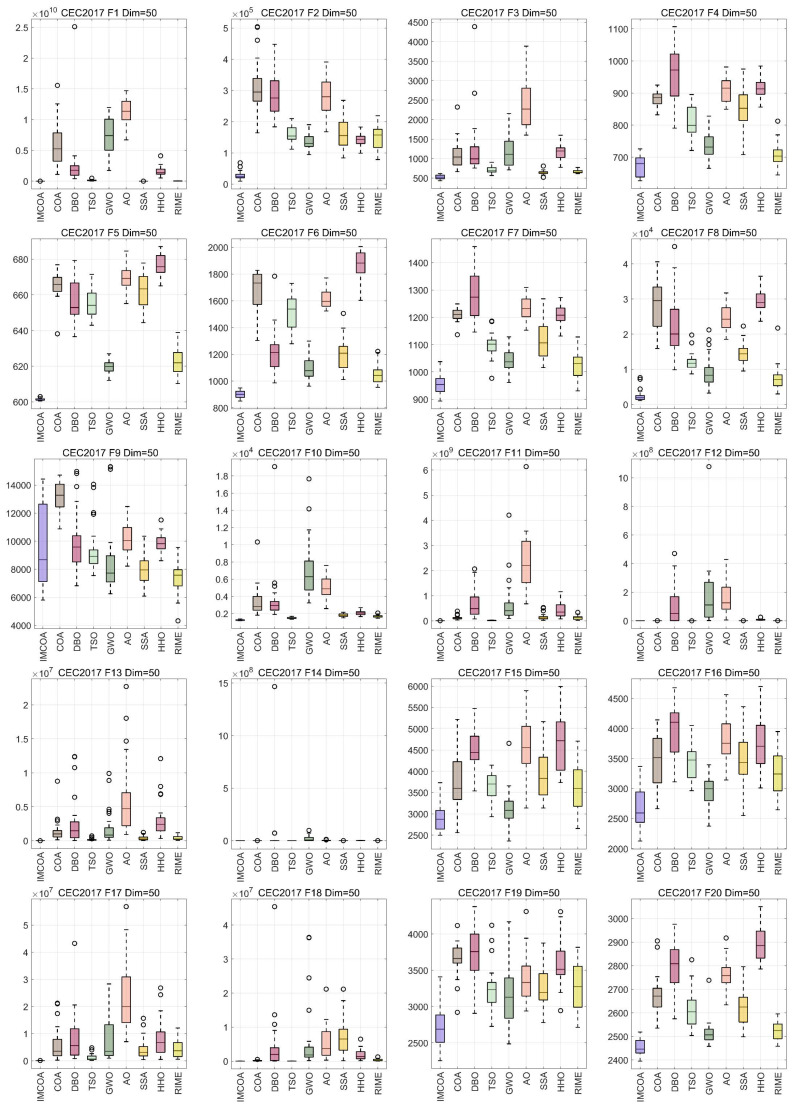

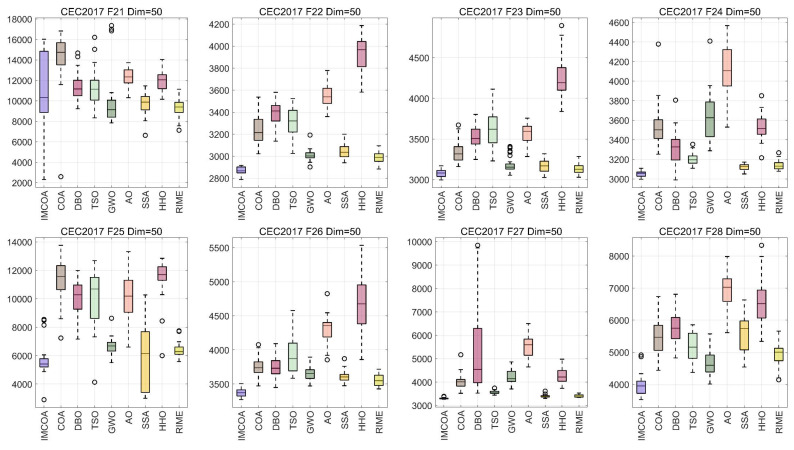
Comparison of box diagrams of the 9 algorithms using CEC2017(Dim = 50).

The convergence curves and box plots of the IMCOA and comparison algorithms solving the CEC2017 test suite of dimensions 50 are shown in Figure A3 and Figure A4 in Appendix A. From Figure 3, it can be seen that IMCOA has better convergence accuracy and faster convergence for all functions except F9 and F21. This indicates that the convergence performance of IMCOA is better than the comparison algorithms. In addition, comparing the convergence in 30 and 50 dimensions, it is shown that the performance of IMCOA continues to provide good optimization results, which indicates that the performance of the improved strategy proposed in this paper does not suffer from an increase in dimensionality.

The results of the box plots illustrate that IMCOA can yield solutions with a more centralized distribution and that these solutions have better quality. This suggests that IMCOA has excellent performance in global exploration and local development and can consistently provide satisfactory solutions.

##### Statistical Analysis

The results of both statistical tests are given in Table 6 and Table 7. IMCOA shows no differences to any of the algorithms except to RIME for F9. For F21, IMCOA differs from the rest of the algorithms except AO and RIME. With respect to F22, there is no difference between IMCOA and SSA. Other than that, IMCOA is significantly different from the other algorithms for the rest of the functions.

Overall, IMCOA significantly outperforms the comparison algorithm in 26 functions. Especially in comparison to COA, IMCOA’s performance is not weaker than COA in any situation, which indicates that the proposed improvement method does not diminish the performance of COA.

#### 4.2.4. Case 3: CEC2017 (Dim = 100)

##### Quantitative Analysis

**Figure 4:**
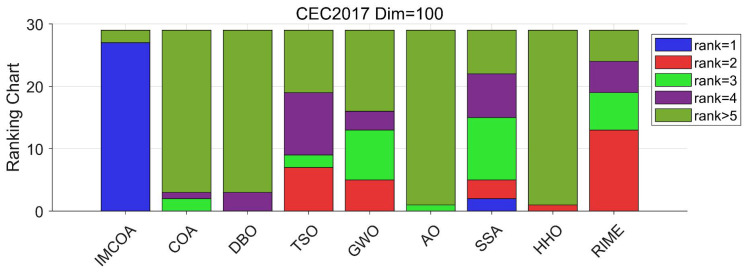
CEC2017 ranking stack chart (Dim = 100).

The results of this experimental case are recorded in Table A4 in Appendix A. IMCOA achieved the best results for all functions except F9 and F21. SSA performed best in F9 and F21. The rest of the algorithms were unable to solve satisfactory results in all the functions in this dimension. Notably, IMCOA achieved 27 first places with an average ranking of 1.38, and COA has a ranking of 6.10. This indicates that the performance of the IMCOA proposed in this paper is significantly boosted. Analyzing the first two cases together, it can be seen that increasing the number of dimensions to 50 will not diminish the performance of IMCOA. When the number of dimensions is further increased to 100, the average ranking of IMCOA does not change much compared with other algorithms, which fully validates the superiority of IMCOA. This shows that the improvement strategy proposed in this paper can effectively improve the solving ability of COA and does not weaken the performance due to the increase in the number of dimensions. Figure 4 shows the ranking of each algorithm when the dimension is 100.

**Figure A5:**
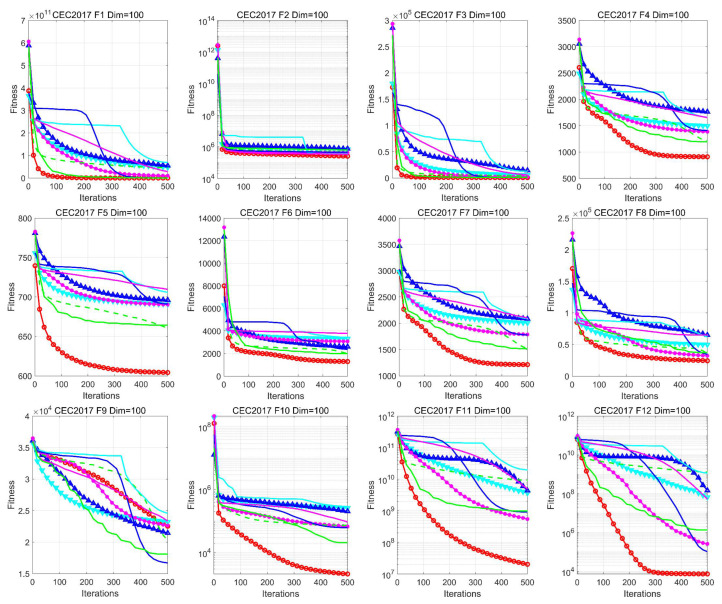

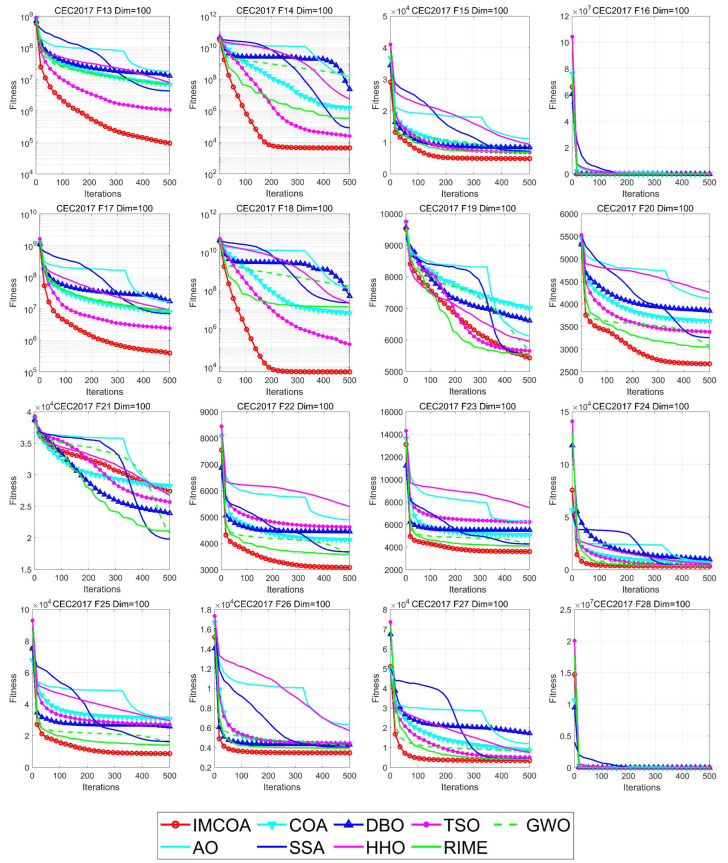
Comparison of convergence curves of the 9 algorithms using CEC2017(Dim = 100).

**Figure A6:**
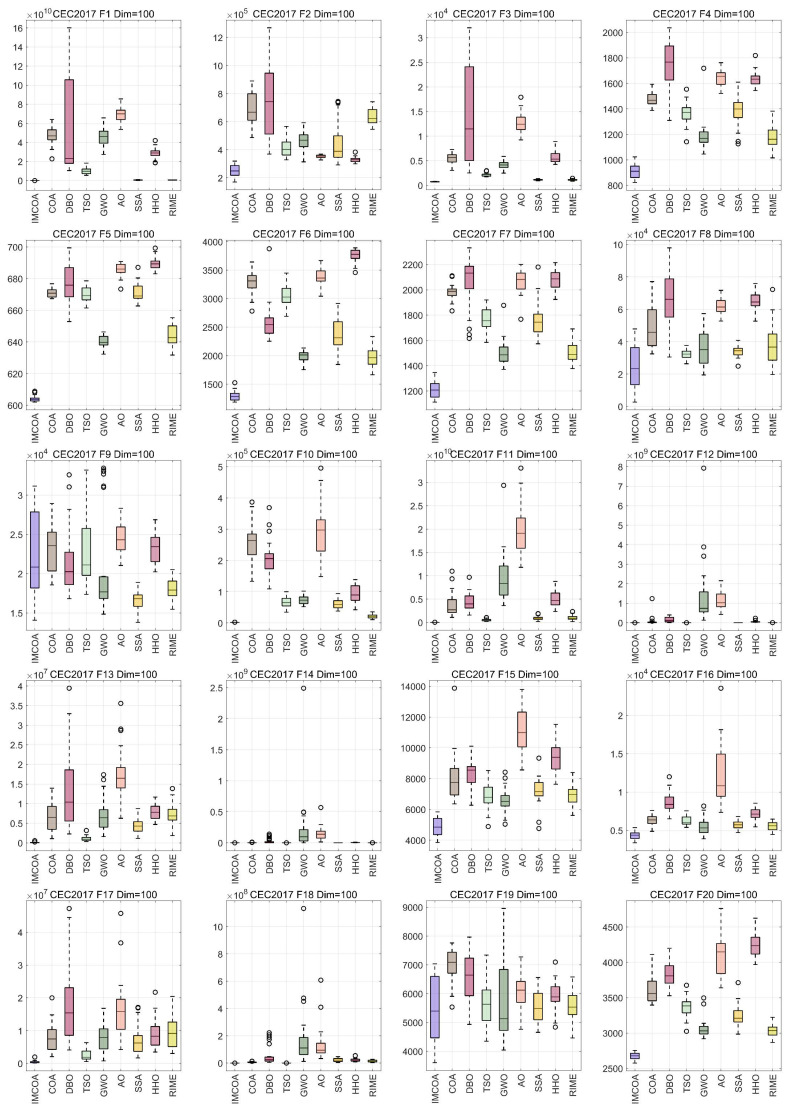

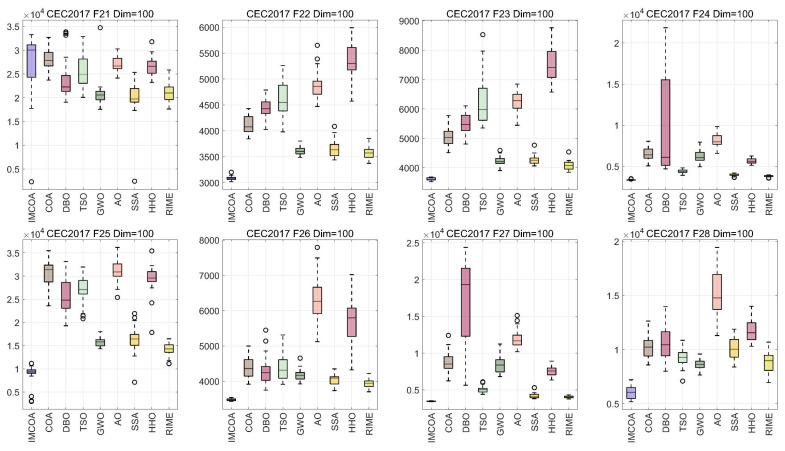
Comparison of the box diagrams of 9 algorithms using CEC2017(Dim = 100).

Figure A5 in Appendix A provides the convergence curves of IMCOA and the comparison algorithm solving the CEC2017 test suite with dimension 100. It can be seen that IMCOA converges faster and with better accuracy in the remaining functions other than F9 and F21, which indicates that IMCOA outperforms the rest of the algorithms in terms of convergence performance. SSA shows better convergence performance for F9 and F21. The quality of the solutions provided by the algorithms involved in the experiment is analyzed using box plots in Figure A6 in Appendix A. Obviously, the solutions of IMCOA have a more centralized distribution and fewer bad values, which indicates the better robustness of IMCOA.

##### Statistical Analysis

The results of the Wilcoxon rank–sum test and the Friedman test are presented in Table 8 and Table 9. IMCOA shows no difference with most algorithms for F9 and F19. There is no significant difference between IMCOA and COA in solving F21. Other than these, there are differences between IMCOA and the rest of the algorithms. Overall, IMCOA was able to outperform all the compared algorithms in 26 functions. IMCOA’s average ranking improved by five places to the first place compared with COA. These data show that IMCOA outperforms the other algorithms on the CEC2017 test suite (100 dimensions). Looking at the statistics of the three dimensions as a whole, IMCOA’s performance is stable in the first place, with significant differences from the other algorithms in some functions. The statistical tests in different dimensions further prove the superior performance of the IMCOA algorithm.

### 4.3. CEC2022 Benchmark Function Experiment

#### 4.3.1. Description of Benchmark Functions

In this section, we further evaluate the performance of IMCOA using the CEC2022 test suite. The basic content of the CEC2022 test suite are shown in Table A5 in Appendix A**.**

#### 4.3.2. Quantitative Analysis

**Figure 5:**
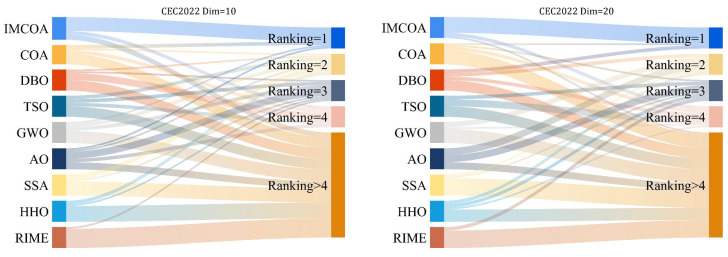
CEC2022 Sankey ranking diagram.

**Figure A7:**
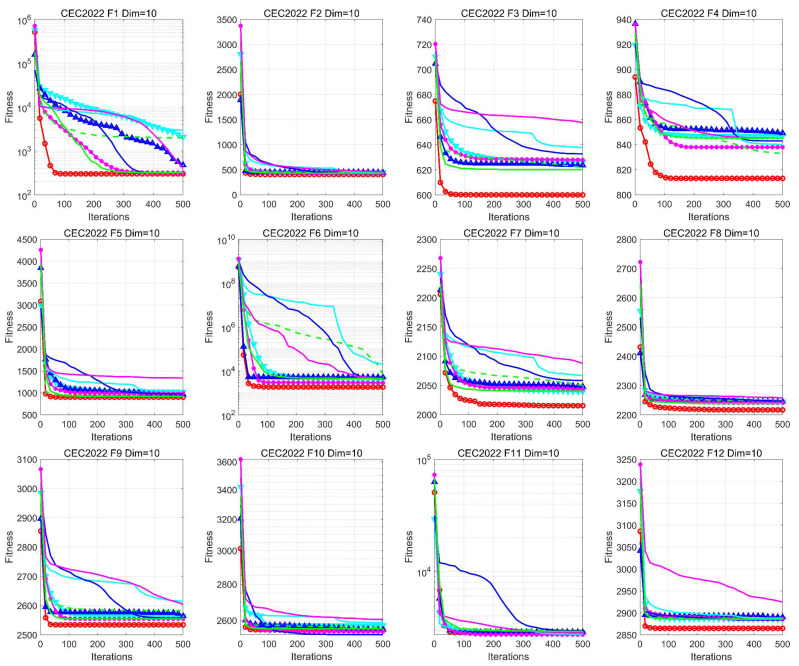

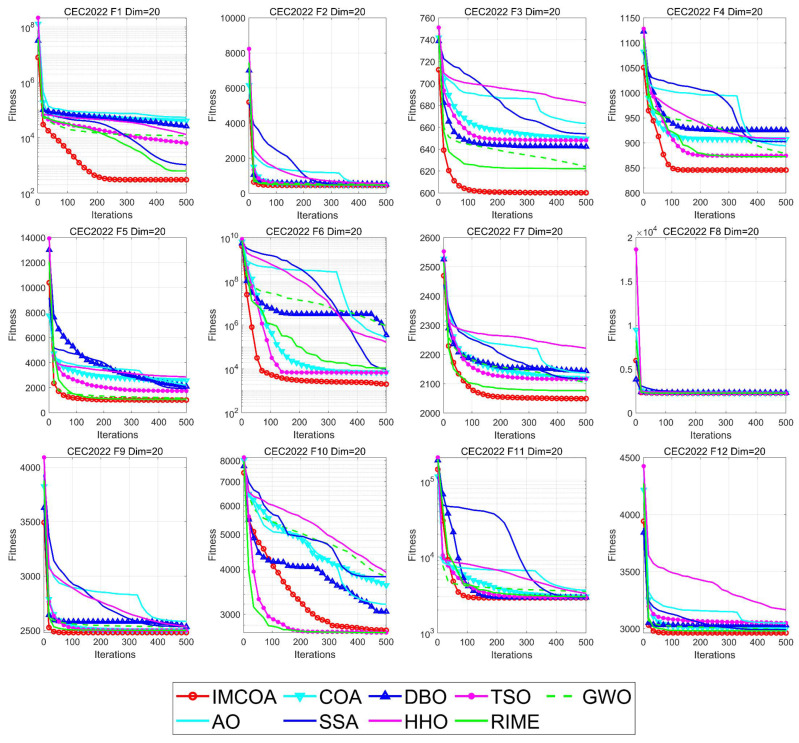
Comparison of convergence curves of the 9 algorithms using CEC2022.

**Figure A8:**
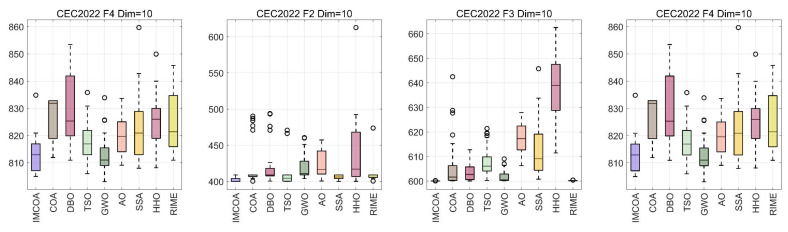

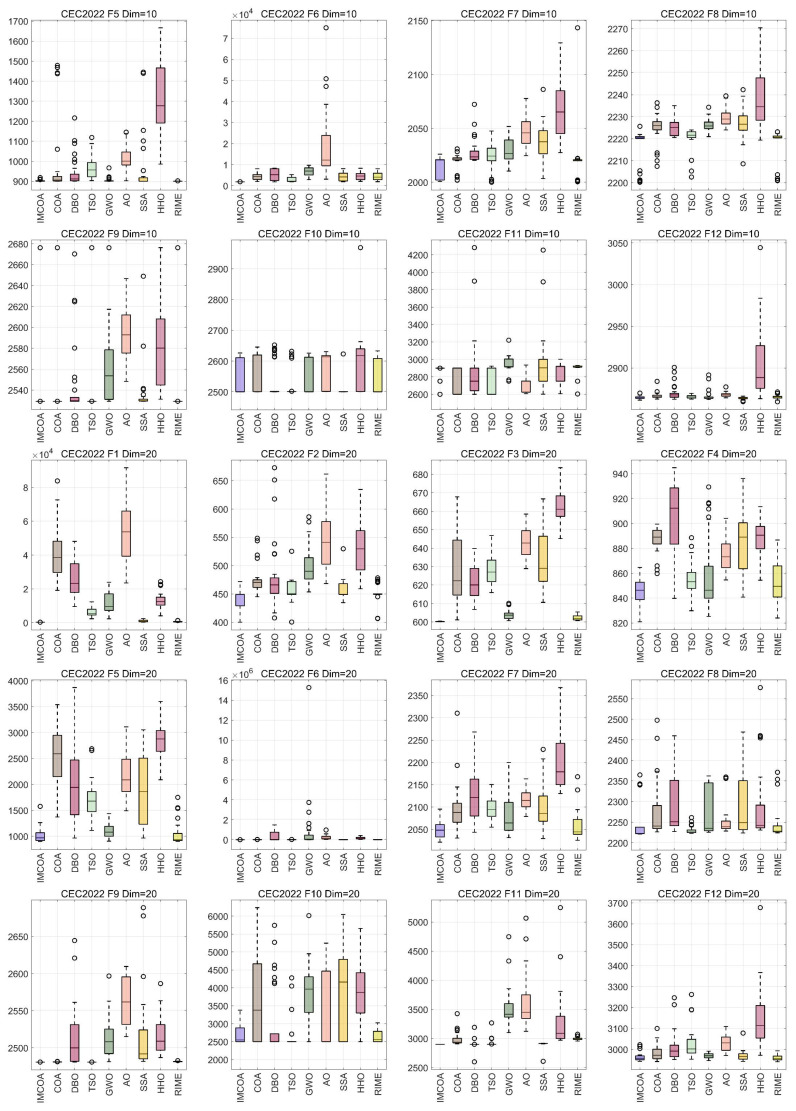
Comparison of box diagrams of the 9 algorithms using CEC2022.

To verify the performance of IMCOA more comprehensively, we validated it in 10 and 20 dimensions of the CEC2022 test suite, respectively. Table A6 and Table A7 show the optimum (Best), average (Ave), standard deviation (std), and ranking of IMCOA and its comparison algorithms for 30 independent runs using different dimensions. Figure 5 plots the Mulberry diagram based on the specific performance of each algorithm in both dimensions. These results show that the IMCOA algorithm proposed in this paper achieves the most significant victory with the first overall ranking. In addition, the convergence curve is shown in Figure A7, which shows that IMCOA is still improving as it has not reached the global optimum. The quality of the solutions of the nine algorithms for the two dimensions of the CEC2022 test suite is comprehensively illustrated in Figure A8 via box plots, and it can be seen that IMCOA achieves excellent results. This proves that the algorithm has excellent global exploration and local utilization capabilities and also proves the effectiveness and accuracy of the IMCOA algorithm proposed in this paper.

#### 4.3.3. Statistical Analysis

The results of the Wilcoxon rank–sum test and Friedman test are given in Table 10, Table 11 and Table 12. Bolded data indicate a *p*-value greater than 0.05. The analysis shows that IMCOA is significantly different from the compared algorithms, except RIME, in most functions. Both IMCOA and RIME are significantly different in seven functions and are not different in five functions. Overall, IMCOA has the best performance, ranking first in both dimensions, with TSO and RIME each achieving second place in one dimension. COA ranks fifth and sixth, respectively. This indicates that the IMCOA proposed in this paper has a significant performance improvement over COA. Furthermore, in terms of overall performance, the decrease in the bolded entries in Table 10 and Table 11 as the dimensionality increases suggests that the difference between IMCOA and the rest of the algorithms will be more pronounced when solving in 20 dimensions, corroborating the potential of IMCOA to solve high-dimensional optimization problems. The superior performance of IMCOA is also confirmed by the fact that the number of “+” values is more than the number of “−” values in Table 12.

### 4.4. Impact Analysis of the Improved Strategies

In this section, we verify the effectiveness of each improvement strategy. The following four strategies are proposed in this paper: cave candidacy strategy, fitness–distance balanced competition strategy, food covariance learning strategy and optimal non-monopoly strategy. In order to analyze the four strategies together, the algorithm combining the cave candidacy strategy and the fitness–distance balanced competition strategy was named IMCOA-1, considering that both the heat avoidance and competition phases were conducted at temperatures greater than 30. The algorithm combining the food covariance learning strategy was named IMCOA-2, and the algorithm combining the optimal non-monopoly search strategy was named IMCOA-3. The three derived algorithms, COA, and IMCOA containing all four strategies were tested using the CEC2017 test function in 3 dimensions (30, 50 and 100) and the CEC2022 test suite in 2 dimensions (10 and 20). The parameter settings were kept constant.

**Figure 6:**
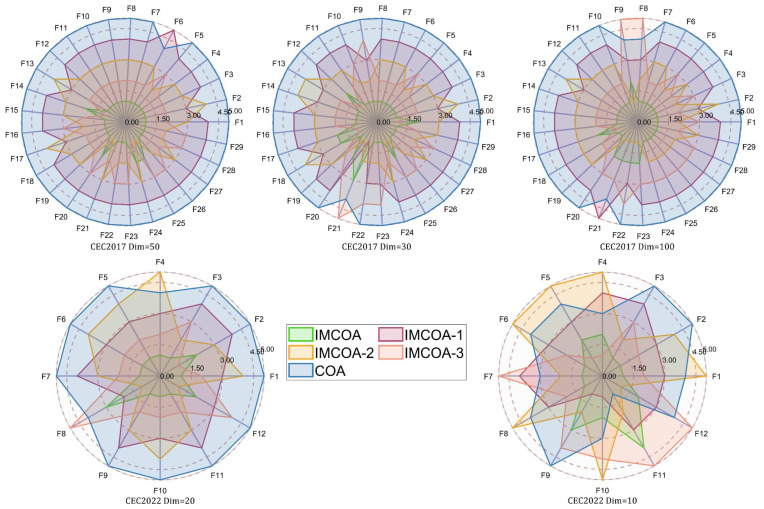
Ranking of each strategy on the CEC2017 and CEC2022 test suites.

Table 13 records the results of the Friedman test for the algorithms involved in the experiment in the five sets of tests. It can be seen that IMCOA, which contains all the strategies, has the best performance, as opposed to COA, which has the worst overall performance. The performance of the three derived algorithms in descending order is IMCOA-3 > IMCOA-2 > IMCOA-1. In order to visualize the performance of these algorithms, radar plots are drawn based on the rankings, as shown in Figure 6. The size of the area enclosed by each curve represents the good or poor performance of this algorithm. The smaller area represents the better performance of this algorithm. We can see that IMCOA has the smallest area surrounded by the curve, which indicates that IMCOA has the best performance. Overall, this algorithm shows good optimization performance in different dimensions, which indicates that our three proposed strategies effectively improve the performance of COA. Specifically, the optimal non-monopoly search strategy perturbs the optimal individuals sufficiently, which greatly improves the ability of the algorithm to get rid of localization and facilitates the further development of COA. The food covariance learning strategy guides the population toward the correct direction of evolution and enriches the population diversity. The cave candidacy strategy and fitness–distance balanced competition strategy balance well the development and exploration performance of COA and enhance the performance of COA.

## 5. Experimental Results and Discussion of Engineering Optimization Problems

### 5.1. Welded Beam Design Problem

**Figure 7:**
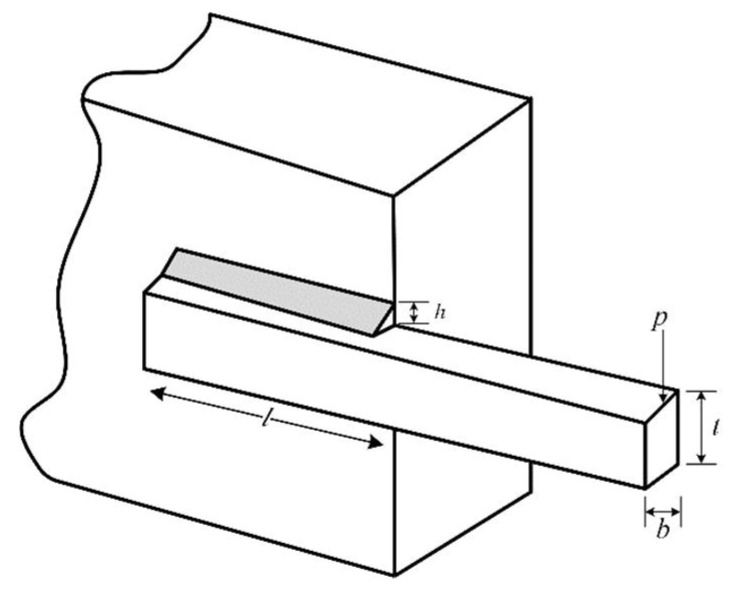
Schematic design of the welded beam.

The objective of the welded beam design problem is to minimize the manufacturing cost of the welded beam [46]. It involves four variables (h, l, t, b) and seven constraints, including tip detection, weld coverage area, bending stress, buckling load, and manufacturing cost. The schematic diagram for this engineering design problem is illustrated in Figure 7. The mathematical formulation for this engineering design problem is given below:

Consider

(26) $$x=[x_{1}\;x_{2}\;x_{3}\;x_{4}]=[h\;l\;t\;b]$$

Minimize

(27) $$f(x)=1.10471x_{1}^{2}x_{2}+0.04811x_{3}x_{4}(14.0+x_{2})$$

Variable range:

(28) $$0.1\leq x_{i}\leq 2,i=1,4;0.1\leq x_{i}\leq 10,i=2.3$$

Subject to

$$\begin{array}{c} g_{1}\left(\vec{x}\right)=\tau \left(\vec{x}\right)-\tau_{\max}\leq 0,g_{2}\left(\vec{x}\right)=\sigma \left(\vec{x}\right)-\sigma_{\max}\leq 0,g_{3}\left(\vec{x}\right)=\delta \left(\vec{x}\right)-\delta_{\max}\leq 0\\ g_{4}\left(\vec{x}\right)=x_{1}-x_{4}\leq 0,g_{5}\left(\vec{x}\right)=P-P_{c}\left(\vec{x}\right)\leq 0,g_{6}\left(\vec{x}\right)=0.125-x_{1}\leq 0\\ g_{7}\left(\vec{x}\right)=1.10471x_{1}^{2}+0.04811x_{3}x_{4}\left(14.0+x_{2}\right)-0.5\leq 0 \end{array}$$

where

$$\begin{array}{c} \tau \left(\vec{x}\right)=\sqrt{{\left(\tau^{\prime }\right)}^{2}+2\tau^{\prime }\tau^{"}\frac{x_{2}}{2R}+\left(\tau^{"}\right)},\tau^{\prime }=\frac{P}{\sqrt{2x_{1}x_{2}}},\tau^{"}=\frac{MR}{J},M=P\left(L+\frac{x_{2}}{2}\right)\\ J=2\left\{\sqrt{2x_{1}x_{2}}\left[\frac{x_{x}^{2}}{4}+{\left(\frac{x_{1}+x_{3}}{2}\right)}^{2}\right]\right\},\delta \left(\vec{x}\right)=\frac{6PL^{3}}{Ex_{4}x_{3}^{2}},R=\sqrt{\frac{x_{2}^{2}}{4}+{\left(\frac{x_{1}+x_{3}}{2}\right)}^{2}},\sigma \left(\vec{x}\right)=\frac{6PL}{x_{4}x_{3}^{2}}\\ P_{c}\left(\vec{x}\right)=\frac{\sqrt[{4.013E}]{\frac{x_{3}^{2}x_{4}^{6}}{0}}}{L^{2}},\left(1-\frac{x_{3}}{2L}\sqrt{\frac{E}{4G}}\right),\left(1-\frac{x_{3}}{2L}\sqrt{\frac{E}{4G}}\right)\\ P=6000lb,L=14,\delta_{\max}=0.25,E=30\times 10^{6}psi,\\ \tau_{\max}=13,600\;\;\;\;\;psi,and\;\;\;\;\;\sigma_{\max}=30,000\;\;\;\;\;\;\;psi \end{array}$$

IMCOA and the competitors in Section 4.1 are applied to the welding beam problem for comparison. According to the results in Table 14, it is found that the minimum production cost, which is 1.692807, can be obtained by using IMCOA.

### 5.2. Tension/Compression Spring Design Problem

**Figure 8:**
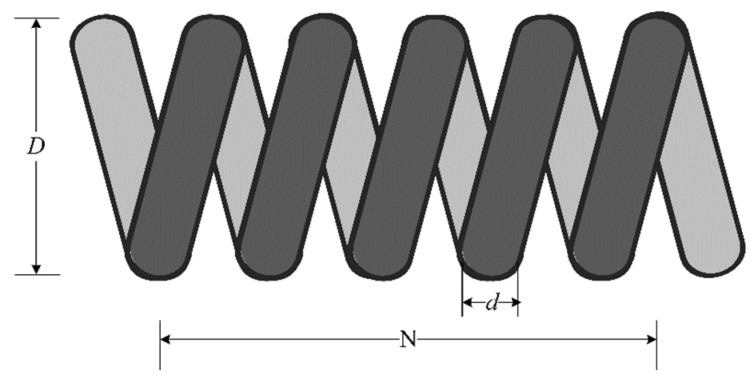
Schematic design of the tension/compression spring design problem.

The tension/compression spring design is an optimization problem in engineering science aimed at reducing the weight of tension/compression springs [47]. It has four design factors that need to be optimized as shown in Figure 8, and four restrictions that should be considered. The primary objective of this problem is to minimize the weight of the spring by selecting three variables: wire diameter (d), mean coil diameter (D), and number of active coils (N). The mathematical formulation for this engineering design problem is given by Equations (29)–(31). The optimal weight of the compression spring derived by the proposed IMCOA is 0.010614. Table 15 demonstrates that all variants of the proposed algorithm outperform all other algorithms in terms of the optimum value. This implies that all variants of the proposed IMCOA are more effective than other competitors in solving this problem.

Consider

(29) $$x=[x_{1}\;x_{2}\;x_{3}]=[d\;D\;N]$$

Minimize

(30) $$f(x)=(x_{3}+2)\times x_{2}\times x_{1}^{2}$$

Variable range:

(31) $$0.05\leq x_{1}\leq 2.0,0.25\leq x_{2}\leq 1.3,2.0\leq x_{3}\leq 15.0$$

Subject to

$$\begin{array}{c} g_{1}(x)=1-\frac{x_{3}\times x_{2}^{3}}{71785\times x_{1}^{4}}\leq 0,g_{2}(x)=\frac{4\times x_{2}^{2}-x_{1}\times x_{2}}{12566\times x_{1}^{4}}+\frac{1}{5108\times x_{1}^{2}}-1\leq 0\\ g_{3}(x)=1-\frac{140.45\times x_{1}}{x_{2}^{2}\times x_{3}}\leq 0,g_{4}(x)=\frac{x_{1}+x_{2}}{1.5}-1\leq 0 \end{array}$$

### 5.3. Pressure Vessel Design Problem

**Figure 9:**
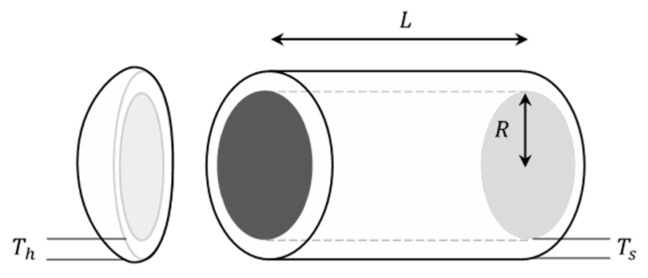
Schematic design of the pressure vessel design problem.

The major aims of this design is to optimize the cost of materials in the formation and welding of a vessel, which is one of the common complex engineering-type problems [48]. This problem, as shown in Figure 9, contains four parametric conditions: the thickness of the shell (Ts), the thickness of the head (Th), the inner radius (R), and the length of the cylindrical section of the vessel (L). The mathematical model is developed with the simultaneous satisfaction of the four constraints as shown below:

Consider

(32) $$\vec{x}=\left[x_{1}\;\;\;x_{2}\;\;\;x_{3}\;\;\;x_{4}\right]=\left[T_{s}\;\;\;T_{h}\;\;\;R\;\;\;L\right]$$

Minimize

(33) $$f\left(\vec{x}\right)=0.6224x_{1}x_{2}x_{3}+1.7781x_{2}x_{3}^{2}+3.1661x_{1}^{2}x_{4}+19.84x_{1}^{2}x_{3}$$

Variable range:

(34) $$0\leq x_{1}\leq 99,0\leq x_{2}\leq 99,10\leq x_{3}\leq 200,10\leq x_{4}\leq 200$$

Subject to

$$\begin{array}{c} g_{1}(\vec{x})=-x_{1}+0.0193x_{3}\leq 0,g_{2}(\vec{x})=-x_{3}+0.00954x_{3}\leq 0\\ g_{3}(\vec{x})=-\pi x_{3}^{2}x_{4}+\frac{4}{3}\pi x_{3}^{3}+1,296,000\leq 0,g_{4}(\vec{x})=-x_{4}-240\leq 0 \end{array}$$

IMCOA was compared with other algorithms on this problem model, and the results are shown in Table 16. The comparison clearly shows that IMCOA outperforms the competitors with the lowest cost of 5744.48400, which indicates that the algorithm has good optimization ability in this type of problem and can improve the design parameters effectively.

## 6. Conclusions

Aimed at addressing the shortcomings of the crayfish optimization algorithm such as its slow convergence speed, ease of falling into the local optimum, and low convergence accuracy, an improved multi-strategy crayfish optimization algorithm (IMCOA) is proposed. In IMCOA, a cave candidacy strategy and a fitness–distance balanced competition strategy are used to balance the development and exploration capabilities of the heat avoidance and competition phases to prevent the algorithm from falling into the local optimum. We propose using the food covariance learning strategy to improve the algorithm’s search efficiency, convergence speed, and robustness. By using the optimal non-monopoly search strategy for the optimal solution, the discovery of high-quality solutions is enhanced, and the optimization capability is improved. We evaluated the performance of IMCOA using 41 test functions from the CEC2017 and CEC2022 test suites, demonstrating its effectiveness on test functions in different dimensions. Using Wilcoxon and Friedman test statistical analyses, we confirm the significant advantages of IMCOA over its competitors in 27, 26, or 26 CEC2017 functions (Dim = 30, 50, or 100) and in 7 and 6 CEC2022 functions (Dim = 10 and 20). Additionally, we validated the applicability of IMCOA in three engineering design optimization problems, including the welded beam design problem, the tension/compression spring design problem, and the pressure vessel design problem. The experimental results thoroughly confirmed the practicality and outstanding performance of the IMCOA across the three optimization problems.

Next, we will continue to explore the applications of IMCOA in other areas, such as image segmentation, intrusion detection, wireless sensor coverage, and vehicle scheduling problems. Meanwhile, inspired by the literature [49], we can combine the characteristics of distributed algorithms to develop a distributed crayfish algorithm. In addition, multi-objective and constrained optimization problems become more important as real-world problems become more complex. Future research will develop a multi-objective version of IMCOA, focusing on IMCOA’s ability to solve multi-objective problems and to provide more comprehensive solutions to optimization problems.

## Figures and Tables

**Table 1:** Metaheuristic algorithms.

Evolution-based algorithms	Genetic Algorithm (GA) [14]
	Differential Evolution (DE) [15]
	Genetic Programming (GP) [16]
	Evolutionary Strategy (ES) [17]
Physics-based algorithms	Simulated Annealing (SA) [18]
	Gravitational Search Algorithm (GSA) [19]
	Sine Cosine Algorithm (SCA) [20]
	Multi-Verse Optimization (MVO) [21]
	Henry Gas Solubility Optimization (HGSO) [22]
	Snow Ablation Optimizer (SAO) [23]
Human-based algorithms	Teaching- And Learning-Based Optimization (TLBO) [24]
	Social Network Search (SNS) [25]
	Group Teaching Optimization Algorithm (GTOA) [26]
	Running City Game Optimizer (RCGO) [27]
	Social Evolution And Learning Optimization (SELO) [28]
Swarm-based algorithms	Particle Swarm Optimization (PSO) [29]
	Ant Colony Optimization (ACO) [30]
	Whale Optimization Algorithm (WOA) [31]
	Grey Wolf Optimization (GWO) [32]
	Remora Optimization Algorithm (ROA) [33]
	Reptile Search Algorithm (RSA) [34]
	Tuna Swarm Optimization (TSO) [35]
	Sand Cat Swarm Optimization (SCSO) [36]

**Table 2:** Software and hardware for all experiments.

	Item	Description
Software	Language	MATLAB 2020a
	Operating System	Windows 10
Hardware	Hard Drive	1000 GB
	RAM	16.00 GB
	Frequency	2.50 GHz
	CPU	Intel(R) Core(TM) i7-11700

**Table 3:** Parameter settings of each algorithm.

Algorithm	Parameter Setting
IMCOA	k = 1
COA	k = 1
DBO [41]	*p* = 0.2
TSO [35]	c = 0.003
GWO [32]	a = [2, 0]
AO [42]	Alpha = 0.1, delta = 0.1
SSA [43]	no parameters
HHO [44]	E0 ϵ [−1, 1], E1 ϵ [0, 2]
RIME [45]	w = 0.5

**Table 4:** Wilcoxon rank–sum test on CEC2017 (Dim = 30).

	COA	DBO	TSO	GWO	AO	SSA	HHO	RIME
F1	3.02E-11	3.02E-11	3.02E-11	3.02E-11	3.02E-11	8.10E-10	3.02E-11	3.02E-11
F2	3.02E-11	3.02E-11	3.02E-11	3.02E-11	3.02E-11	3.02E-11	3.02E-11	3.02E-11
F3	1.55E-09	1.21E-10	9.83E-08	3.02E-11	3.02E-11	5.46E-06	3.02E-11	2.03E-07
F4	1.46E-10	4.50E-11	3.49E-09	7.73E-06	3.69E-11	3.49E-09	3.02E-11	2.61E-02
F5	3.02E-11	3.02E-11	3.02E-11	3.02E-11	3.02E-11	3.02E-11	3.02E-11	3.02E-11
F6	3.02E-11	3.69E-11	3.02E-11	2.03E-09	3.02E-11	5.49E-11	3.02E-11	2.00E-06
F7	3.02E-11	3.02E-11	5.07E-10	4.98E-04	3.02E-11	8.15E-11	3.02E-11	3.67E-03
F8	3.02E-11	3.02E-11	4.08E-11	8.35E-08	3.02E-11	3.02E-11	3.02E-11	3.32E-06
F9	5.83E-03	2.81E-02	4.51E-02	2.12E-01	6.67E-03	5.11E-01	3.67E-03	4.68E-02
F10	3.02E-11	3.02E-11	1.56E-08	3.02E-11	3.02E-11	1.61E-10	3.02E-11	7.39E-11
F11	3.02E-11	3.69E-11	8.15E-11	3.02E-11	3.02E-11	3.02E-11	3.02E-11	3.02E-11
F12	2.44E-09	3.47E-10	2.84E-01	3.02E-11	3.02E-11	2.87E-10	3.02E-11	7.38E-10
F13	3.02E-11	3.02E-11	3.02E-11	3.02E-11	3.02E-11	3.02E-11	3.02E-11	3.02E-11
F14	3.02E-11	3.02E-11	7.37E-10	3.02E-11	3.02E-11	3.02E-11	3.02E-11	3.02E-11
F15	6.53E-08	4.20E-10	7.20E-05	5.57E-03	1.96E-10	8.15E-05	1.96E-10	1.17E-03
F16	2.32E-06	1.86E-09	7.69E-08	2.15E-02	2.03E-09	1.17E-05	1.96E-10	3.01E-04
F17	3.02E-11	3.02E-11	3.34E-11	3.02E-11	3.02E-11	3.02E-11	3.02E-11	3.02E-11
F18	2.37E-10	7.39E-11	8.89E-10	3.02E-11	3.02E-11	3.02E-11	3.02E-11	2.15E-10
F19	3.83E-06	9.06E-08	2.77E-05	2.32E-02	2.00E-06	9.51E-06	7.12E-09	1.44E-02
F20	2.61E-10	3.02E-11	8.89E-10	2.71E-02	4.08E-11	5.46E-06	3.02E-11	4.86E-03
F21	2.62E-03	2.88E-06	7.73E-02	3.16E-05	5.55E-02	1.33E-02	6.01E-08	3.37E-04
F22	4.50E-11	3.02E-11	3.02E-11	3.35E-08	3.02E-11	5.00E-09	3.02E-11	1.61E-10
F23	8.10E-10	3.02E-11	4.08E-11	1.89E-04	3.02E-11	1.75E-05	3.02E-11	1.04E-04
F24	1.77E-10	1.29E-09	4.12E-06	5.49E-11	3.02E-11	2.44E-09	3.02E-11	4.31E-08
F25	3.37E-05	9.76E-10	3.26E-07	5.86E-06	8.29E-06	9.52E-04	1.69E-09	5.09E-06
F26	1.85E-08	9.26E-09	1.17E-09	4.44E-07	3.02E-11	2.00E-05	3.02E-11	1.09E-01
F27	3.02E-11	3.02E-11	1.21E-10	3.02E-11	3.02E-11	7.39E-11	3.02E-11	4.98E-11
F28	1.09E-10	8.15E-11	2.87E-10	1.07E-09	3.02E-11	3.34E-11	3.02E-11	5.97E-09
F29	3.02E-11	4.50E-11	1.31E-08	3.02E-11	3.02E-11	3.02E-11	3.02E-11	3.02E-11

**Table 5:** Statistical results using CEC2017 (Dim = 30).

Dimensions	30		
Algorithm	Average Rank	Overall Rank	(+/=/−)
IMCOA	1.21	1	
COA	5.62	6	29/0/0
DBO	6.86	7	29/0/0
TSO	3.76	3	27/2/0
GWO	4.76	5	28/1/0
AO	7.79	9	28/1/0
SSA	4.28	4	28/1/0
HHO	7.62	8	29/0/0
RIME	3.10	2	27/1/1

**Table 6:** Wilcoxon rank–sum test on CEC2017 (Dim = 50).

	COA	DBO	TSO	GWO	AO	SSA	HHO	RIME
F1	3.02E-11	3.02E-11	3.02E-11	3.02E-11	3.02E-11	3.02E-11	3.02E-11	3.02E-11
F2	3.02E-11	3.02E-11	3.02E-11	3.02E-11	3.02E-11	3.02E-11	3.02E-11	3.02E-11
F3	3.02E-11	3.02E-11	2.15E-10	3.02E-11	3.02E-11	8.89E-10	3.02E-11	3.34E-11
F4	3.02E-11	3.02E-11	4.08E-11	1.47E-07	3.02E-11	4.98E-11	3.02E-11	8.56E-04
F5	3.02E-11	3.02E-11	3.02E-11	3.02E-11	3.02E-11	3.02E-11	3.02E-11	3.02E-11
F6	3.02E-11	3.02E-11	3.02E-11	3.02E-11	3.02E-11	3.02E-11	3.02E-11	3.02E-11
F7	3.02E-11	3.02E-11	5.49E-11	5.00E-09	3.02E-11	5.49E-11	3.02E-11	9.53E-07
F8	3.02E-11	3.02E-11	3.02E-11	5.07E-10	3.02E-11	3.02E-11	3.02E-11	2.92E-09
F9	3.83E-06	**3.55E-01**	**6.95E-01**	**1.91E-01**	**2.71E-01**	**7.24E-02**	**3.04E-01**	7.29E-03
F10	3.02E-11	3.02E-11	6.07E-11	3.02E-11	3.02E-11	3.02E-11	3.02E-11	3.02E-11
F11	3.02E-11	3.02E-11	3.02E-11	3.02E-11	3.02E-11	3.02E-11	3.02E-11	3.02E-11
F12	3.02E-11	3.02E-11	3.01E-07	3.02E-11	3.02E-11	3.02E-11	3.02E-11	3.02E-11
F13	3.02E-11	3.02E-11	3.02E-11	3.02E-11	3.02E-11	3.02E-11	3.02E-11	3.02E-11
F14	6.07E-11	3.02E-11	1.22E-02	3.02E-11	3.02E-11	6.07E-11	3.02E-11	3.02E-11
F15	4.11E-07	3.69E-11	2.92E-09	2.15E-02	6.70E-11	5.57E-10	3.02E-11	2.57E-07
F16	2.02E-08	4.50E-11	4.20E-10	6.55E-04	4.98E-11	3.20E-09	1.78E-10	1.25E-07
F17	3.02E-11	3.02E-11	3.02E-11	3.02E-11	3.02E-11	3.02E-11	3.02E-11	3.02E-11
F18	2.61E-10	1.61E-10	4.43E-03	3.02E-11	3.02E-11	3.02E-11	3.02E-11	3.69E-11
F19	7.39E-11	1.33E-10	8.35E-08	2.43E-05	1.55E-09	2.83E-08	9.92E-11	8.35E-08
F20	3.02E-11	3.02E-11	7.39E-11	4.11E-07	3.02E-11	4.08E-11	3.02E-11	1.25E-07
F21	1.58E-04	**2.97E-01**	**4.83E-01**	**8.24E-02**	3.78E-02	**1.91E-01**	**1.02E-01**	3.51E-02
F22	3.02E-11	3.02E-11	3.02E-11	6.07E-11	3.02E-11	3.02E-11	3.02E-11	3.16E-10
F23	3.34E-11	3.02E-11	3.02E-11	7.60E-07	3.02E-11	4.12E-06	3.02E-11	1.24E-03
F24	3.02E-11	3.82E-09	3.02E-11	3.02E-11	3.02E-11	1.69E-09	3.02E-11	1.96E-10
F25	4.08E-11	6.07E-11	2.23E-09	4.44E-07	1.21E-10	**9.71E-01**	5.49E-11	8.20E-07
F26	4.08E-11	4.98E-11	3.02E-11	5.49E-11	3.02E-11	4.08E-11	3.02E-11	1.07E-09
F27	3.02E-11	3.02E-11	3.02E-11	3.02E-11	3.02E-11	8.48E-09	3.02E-11	5.07E-10
F28	6.70E-11	3.69E-11	1.46E-10	1.56E-08	3.02E-11	6.70E-11	3.02E-11	4.20E-10
F29	3.02E-11	3.02E-11	8.10E-10	3.02E-11	3.02E-11	3.02E-11	3.02E-11	3.02E-11

**Table 7:** Statistical results using CEC2017 (Dim = 50).

Dimensions	50		
Algorithm	Average Rank	Overall Rank	(+/=/−)
IMCOA	1.24	1	
COA	6.03	6	29/0/0
DBO	6.93	7	27/2/0
TSO	3.90	3	27/2/0
GWO	4.76	5	27/2/0
AO	7.93	9	28/1/0
SSA	4.10	4	26/3/0
HHO	7.10	8	27/2/0
RIME	3.00	2	27/0/2

**Table 8:** Wilcoxon rank–sum test on CEC2017 (Dim = 100).

	COA	DBO	TSO	GWO	AO	SSA	HHO	RIME
F1	3.02E-11	3.02E-11	3.02E-11	3.02E-11	3.02E-11	3.02E-11	3.02E-11	3.02E-11
F2	3.02E-11	3.02E-11	3.02E-11	6.07E-11	3.02E-11	7.39E-11	3.82E-10	3.02E-11
F3	3.02E-11	3.02E-11	3.02E-11	3.02E-11	3.02E-11	3.02E-11	3.02E-11	3.02E-11
F4	3.02E-11	3.02E-11	3.02E-11	3.02E-11	3.02E-11	3.02E-11	3.02E-11	3.34E-11
F5	3.02E-11	3.02E-11	3.02E-11	3.02E-11	3.02E-11	3.02E-11	3.02E-11	3.02E-11
F6	3.02E-11	3.02E-11	3.02E-11	3.02E-11	3.02E-11	3.02E-11	3.02E-11	3.02E-11
F7	3.02E-11	3.02E-11	3.02E-11	3.02E-11	3.02E-11	3.02E-11	3.02E-11	3.02E-11
F8	6.01E-08	1.07E-09	2.51E-02	1.00E-03	3.02E-11	5.08E-03	3.02E-11	8.56E-04
F9	**6.31E-01**	**6.41E-01**	**7.62E-01**	**8.77E-02**	**2.46E-01**	1.87E-05	**5.01E-01**	2.38E-03
F10	3.02E-11	3.02E-11	3.02E-11	3.02E-11	3.02E-11	3.02E-11	3.02E-11	3.02E-11
F11	3.02E-11	3.02E-11	3.02E-11	3.02E-11	3.02E-11	3.02E-11	3.02E-11	3.02E-11
F12	3.02E-11	3.02E-11	3.02E-11	3.02E-11	3.02E-11	3.02E-11	3.02E-11	3.02E-11
F13	3.02E-11	3.02E-11	4.50E-11	3.02E-11	3.02E-11	3.02E-11	3.02E-11	3.02E-11
F14	3.02E-11	3.02E-11	2.37E-10	3.02E-11	3.02E-11	3.34E-11	3.02E-11	3.02E-11
F15	3.02E-11	3.02E-11	1.96E-10	1.17E-09	3.02E-11	4.62E-10	3.02E-11	7.39E-11
F16	4.50E-11	3.02E-11	3.02E-11	3.09E-06	3.02E-11	4.62E-10	3.02E-11	2.23E-09
F17	3.02E-11	3.02E-11	3.16E-10	4.08E-11	3.02E-11	3.34E-11	3.02E-11	3.02E-11
F18	3.02E-11	3.02E-11	5.49E-11	3.02E-11	3.02E-11	3.02E-11	3.02E-11	3.02E-11
F19	1.47E-07	2.84E-04	**6.41E-01**	**4.92E-01**	**6.35E-02**	**9.12E-01**	**2.17E-01**	**9.47E-01**
F20	3.02E-11	3.02E-11	3.02E-11	3.02E-11	3.02E-11	3.02E-11	3.02E-11	3.02E-11
F21	**1.71E-01**	1.33E-02	9.47E-03	9.51E-06	5.32E-03	7.60E-07	9.07E-03	7.74E-06
F22	3.02E-11	3.02E-11	3.02E-11	3.02E-11	3.02E-11	3.02E-11	3.02E-11	3.02E-11
F23	3.02E-11	3.02E-11	3.02E-11	3.02E-11	3.02E-11	3.02E-11	3.02E-11	3.02E-11
F24	3.02E-11	3.02E-11	3.02E-11	3.02E-11	3.02E-11	3.02E-11	3.02E-11	3.02E-11
F25	3.02E-11	3.02E-11	3.02E-11	3.02E-11	3.02E-11	3.82E-10	3.02E-11	3.69E-11
F26	3.02E-11	3.02E-11	3.02E-11	3.02E-11	3.02E-11	3.02E-11	3.02E-11	3.02E-11
F27	3.02E-11	3.02E-11	3.02E-11	3.02E-11	3.02E-11	3.02E-11	3.02E-11	3.02E-11
F28	3.02E-11	3.02E-11	3.34E-11	3.02E-11	3.02E-11	3.02E-11	3.02E-11	3.69E-11
F29	3.02E-11	3.02E-11	3.02E-11	3.02E-11	3.02E-11	3.02E-11	3.02E-11	3.02E-11

**Table 9:** Statistical results using CEC2017 (Dim = 100).

Dimensions	100		
Algorithm	Average Rank	Overall Rank	(+/=/−)
IMCOA	1.38	1	
COA	6.10	6	27/2/0
DBO	6.97	8	27/2/1
TSO	4.10	4	26/2/1
GWO	4.59	5	26/2/1
AO	8.10	9	26/2/1
SSA	3.59	3	26/1/2
HHO	6.93	7	26/2/1
RIME	3.24	2	26/1/2

**Table 10:** Wilcoxon rank–sum test using CEC2022 (Dim = 10).

	COA	DBO	TSO	GWO	AO	SSA	HHO	RIME
F1	3.02E-11	3.02E-11	3.02E-11	3.02E-11	3.02E-11	3.02E-11	3.02E-11	3.02E-11
F2	5.08E-08	1.92E-06	5.54E-03	1.28E-09	1.28E-09	8.65E-05	2.67E-06	4.79E-07
F3	7.39E-11	3.47E-10	3.02E-11	8.15E-11	3.02E-11	3.02E-11	3.02E-11	2.92E-09
F4	3.01E-07	1.16E-07	5.82E-03	**9.00E-01**	2.25E-04	9.79E-05	4.11E-07	3.57E-06
F5	3.16E-05	4.80E-07	1.46E-10	1.99E-02	1.33E-10	**9.05E-02**	3.02E-11	**8.42E-01**
F6	3.02E-11	4.98E-11	6.70E-11	3.02E-11	3.02E-11	3.02E-11	3.02E-11	3.02E-11
F7	1.06E-03	7.09E-08	3.56E-04	1.16E-07	3.34E-11	6.12E-10	3.02E-11	**8.30E-01**
F8	2.78E-07	5.53E-08	3.34E-03	2.61E-10	5.49E-11	6.52E-09	2.87E-10	**3.33E-01**
F9	2.11E-10	7.62E-03	**3.01E-01**	2.57E-10	2.83E-10	2.83E-10	2.57E-10	2.57E-10
F10	1.76E-02	**1.19E-01**	**1.30E-01**	**6.52E-01**	6.20E-04	**6.31E-01**	1.32E-04	**4.73E-01**
F11	3.78E-02	1.96E-04	2.13E-04	3.50E-09	1.12E-02	1.27E-02	**5.55E-02**	3.50E-09
F12	5.57E-03	4.21E-04	**1.58E-01**	**4.73E-01**	7.03E-07	**8.23E-02**	1.85E-09	**8.77E-02**

**Table 11:** Wilcoxon rank–sum test using CEC2022 (Dim = 20).

	COA	DBO	TSO	GWO	AO	SSA	HHO	RIME
F1	3.02E-11	3.02E-11	3.02E-11	3.02E-11	3.02E-11	3.02E-11	3.02E-11	3.02E-11
F2	8.48E-09	7.22E-06	3.16E-05	1.61E-10	4.08E-11	1.49E-06	5.49E-11	1.86E-06
F3	3.02E-11	3.02E-11	3.02E-11	3.02E-11	3.02E-11	3.02E-11	3.02E-11	3.02E-11
F4	4.98E-11	3.16E-10	1.50E-02	**4.12E-01**	5.57E-10	9.83E-08	1.21E-10	**1.58E-01**
F5	4.08E-11	6.72E-10	1.21E-10	8.12E-04	3.34E-11	1.31E-08	3.02E-11	**7.96E-01**
F6	5.07E-10	1.33E-10	5.96E-09	3.02E-11	3.02E-11	1.33E-10	3.02E-11	4.07E-11
F7	2.78E-07	2.39E-08	6.52E-09	1.77E-03	6.70E-11	4.80E-07	3.02E-11	**7.73E-01**
F8	5.61E-05	5.86E-06	2.38E-03	6.77E-05	2.84E-04	7.74E-06	4.94E-05	6.55E-04
F9	3.02E-11	3.02E-11	3.02E-11	3.02E-11	3.02E-11	3.02E-11	3.02E-11	3.02E-11
F10	2.01E-04	**1.45E-01**	**7.39E-01**	3.83E-06	**9.33E-02**	4.98E-04	2.39E-08	**8.07E-01**
F11	3.02E-11	7.08E-08	3.02E-11	3.02E-11	3.02E-11	5.57E-10	3.02E-11	3.02E-11
F12	7.62E-03	1.02E-05	2.38E-07	8.68E-03	1.07E-09	**5.75E-02**	1.46E-10	**9.71E-01**

**Table 12:** Statistical results using CEC2022.

Dimensions	10			20		
Algorithm	Average Rank	Overall Rank	(+/=/−)	Average Rank	Overall Rank	(+/=/−)
IMCOA	1.96	1		1.42	1	
COA	5.50	5	11/1/0	5.67	6	12/0/0
DBO	5.75	7	11/0/1	6.25	7	11/1/0
TSO	3.21	2	8/3/1	3.42	3	10/1/1
GWO	5.50	5	9/3/0	5.25	4	11/1/0
AO	7.33	8	11/0/1	7.17	8	11/1/0
SSA	4.33	4	9/3/0	5.33	5	11/1/0
HHO	7.67	9	11/1/0	7.83	9	12/0/0
RIME	3.75	3	7/5/0	2.67	2	6/5/1
Overall (+/=/−)			77/16/3			84/10/2

**Table 13:** Friedman test results of each strategy for the CEC2017 and CEC2022 test suites.

Type	Dim	IMCOA	IMCOA-1	IMCOA-2	IMCOA-3	COA
CEC2017	30	**1.28**	3.66	2.72	2.38	4.97
	50	**1.14**	3.93	2.72	2.24	4.97
	100	**1.21**	3.86	2.41	2.62	4.90
CEC2022	10	**1.75**	2.92	3.58	3.00	3.75
	20	**1.33**	3.33	3.08	2.42	4.83
Average rank		**1.34**	3.54	2.91	2.53	4.68

**Table 14:** Experimental results of the welded beam design.

Algorithm	*h*	*l*	*t*	*b*	Optimal Cost
IMCOA	0.205738	3.234802	9.036458	0.205738	1.692807
COA	0.204039	3.53106	9.027463	0.206147	1.731991
DBO [41]	0.205803	3.230860	9.046927	0.206005	1.696152
TSO [35]	0.205700	3.470500	9.036640	0.205700	1.724900
GWO [32]	0.202601	3.297931	9.044665	0.205690	1.697776
AO [42]	0.205760	3.252909	9.036444	0.205741	1.695358
SSA [43]	0.205415	3.472346	9.03522	0.20116	1.72359
HHO [44]	0.184981	3.958342	9.085891	0.206082	1.737752
RIME [45]	0.205745	3.234747	9.036253	0.205746	1.692831

**Table 15:** Experimental results of the tension/compression spring design problem.

Algorithm	*d*	*D*	*N*	Optimal Cost
IMCOA	0.053799	0.46951	5.81122	0.010614
COA	0.051082	0.34226	12.19919	0.012681
DBO [41]	0.050000	0.31740	14.03486	0.012723
TSO [35]	0.051082	0.34226	12.19919	0.012681
GWO [32]	0.050000	0.31741	14.03205	0.012721
AO [42]	0.051629	0.35528	11.37362	0.012665
SSA [43]	0.052056	0.36562	10.78559	0.012667
HHO [44]	0.057631	0.517254	5.722252	0.013266
RIME [45]	0.0554	0.4526	7.2886	0.012901

**Table 16:** Experimental results of the pressure vessel design problem.

Algorithm	*Ts*	*Th*	*R*	*L*	Optimal Cost
IMCOA	0.7379	0.3736	40.4105	198.8007	5744.4840
COA	1.0705	0.5286	55.3885	60.61546	5962.0081
DBO [41]	0.8125	0.4023	42.0984	176.6367	5949.1354
TSO [35]	0.7985	0.4851	40.3391	199.7284	5895.3175
GWO [32]	0.7793	0.3860	40.3824	199.1725	5890.6776
AO [42]	0.7781	0.3846	40.3207	199.9841	5885.3869
SSA [43]	0.8015	0.3962	41.5312	183.9323	5929.6608
HHO [44]	0.7781	0.3846	40.3196	200.0000	6541.0173
RIME [45]	0.7500	0.4375	40.3471	200.0000	5839.6372

## Data Availability

The data presented in this study are available on request from the corresponding author.

## Appendix A

**Table A1:** CEC2017 test functions.

Type	ID	CEC2017 Function Name	Range	Dimension	*f*_min_
Unimodal	F1	Shifted and Rotated Bent Cigar Function	[−100, 100]	30/50/100	100
	F2	Shifted and Rotated Sum of Different Power Function	[−100, 100]	30/50/100	200
	F3	Shifted and Rotated Zakharov Function	[−100, 100]	30/50/100	300
Multimodal	F4	Shifted and Rotated Rosenbrock’s Function	[−100, 100]	30/50/100	400
	F5	Shifted and Rotated Rastrigin’s Function	[−100, 100]	30/50/100	500
	F6	Shifted and Rotated Expanded Scaffer’s F6 Function	[−100, 100]	30/50/100	600
	F7	Shifted and Rotated Lunacek Bi_Rastrigin Function	[−100, 100]	30/50/100	700
	F8	Shifted and Rotated Non-Continuous Rastrigin’s Function	[−100, 100]	30/50/100	800
	F9	Shifted and Rotated Levy Function	[−100, 100]	30/50/100	900
	F10	Shifted and Rotated Schwefel’s Function	[−100, 100]	30/50/100	1000
Hybrid	F11	Hybrid Function 1 (N = 3)	[−100, 100]	30/50/100	1100
	F12	Hybrid Function 2 (N = 3)	[−100, 100]	30/50/100	1200
	F13	Hybrid Function 3 (N = 3)	[−100, 100]	30/50/100	1300
	F14	Hybrid Function 4 (N = 4)	[−100, 100]	30/50/100	1400
	F15	Hybrid Function 5 (N = 4)	[−100, 100]	30/50/100	1500
	F16	Hybrid Function 6 (N = 4)	[−100, 100]	30/50/100	1600
	F17	Hybrid Function 6 (N = 5)	[−100, 100]	30/50/100	1700
	F18	Hybrid Function 6 (N = 5)	[−100, 100]	30/50/100	1800
	F19	Hybrid Function 6 (N = 5)	[−100, 100]	30/50/100	1900
	F20	Hybrid Function 6 (N = 6)	[−100, 100]	30/50/100	2000
Composition	F21	Composition Function 1 (N = 3)	[−100, 100]	30/50/100	2100
	F22	Composition Function 2 (N = 3)	[−100, 100]	30/50/100	2200
	F23	Composition Function 3 (N = 4)	[−100, 100]	30/50/100	2300
	F24	Composition Function 4 (N = 4)	[−100, 100]	30/50/100	2400
	F25	Composition Function 5 (N = 5)	[−100, 100]	30/50/100	2500
	F26	Composition Function 6 (N = 5)	[−100, 100]	30/50/100	2600
	F27	Composition Function 7 (N = 6)	[−100, 100]	30/50/100	2700
	F28	Composition Function 8 (N = 6)	[−100, 100]	30/50/100	2800
	F29	Composition Function 9 (N = 3)	[−100, 100]	30/50/100	2900
	F30	Composition Function 10 (N = 3)	[−100, 100]	30/50/100	3000

**Table A2:** CEC2017 experimental results (Dim = 30).

Function	Index	IMCOA	COA	DBO	TSO	GWO	AO	SSA	HHO	RIME
F1	Best	1.12E+02	3.64E+06	2.10E+04	5.73E+04	2.49E+08	9.02E+08	1.33E+04	3.35E+07	1.00E+06
	Ave	6.22E+03	2.16E+08	1.14E+08	8.56E+05	1.74E+09	1.93E+09	2.32E+04	8.43E+07	2.42E+06
	Std	6.65E+03	4.46E+08	1.07E+08	1.74E+06	9.78E+08	6.00E+08	5.98E+03	3.72E+07	1.02E+06
	Rank	1	7	6	3	8	9	2	5	4
F2	Best	3.06E+02	4.77E+04	4.98E+04	3.06E+04	3.01E+04	4.25E+04	1.68E+04	2.97E+04	1.50E+04
	Ave	4.67E+02	9.97E+04	8.87E+04	5.09E+04	4.97E+04	5.83E+04	3.34E+04	4.58E+04	2.76E+04
	Std	2.31E+02	2.52E+04	2.17E+04	1.18E+04	1.15E+04	7.20E+03	9.90E+03	8.97E+03	8.95E+03
	Rank	1	9	8	6	5	7	3	4	2
F3	Best	4.00E+02	4.72E+02	4.92E+02	4.05E+02	5.24E+02	5.88E+02	4.70E+02	5.30E+02	4.55E+02
	Ave	4.63E+02	5.46E+02	6.42E+02	5.15E+02	6.04E+02	7.85E+02	5.07E+02	6.03E+02	5.21E+02
	Std	3.95E+01	4.01E+01	1.54E+02	3.06E+01	9.50E+01	1.62E+02	2.58E+01	5.32E+01	2.92E+01
	Rank	1	5	8	3	7	9	2	6	4
F4	Best	5.42E+02	6.12E+02	6.32E+02	5.91E+02	5.80E+02	6.44E+02	5.84E+02	6.80E+02	5.51E+02
	Ave	5.83E+02	7.42E+02	7.15E+02	6.62E+02	6.18E+02	7.18E+02	6.67E+02	7.55E+02	5.98E+02
	Std	2.71E+01	6.11E+01	5.29E+01	4.34E+01	3.54E+01	3.43E+01	5.08E+01	3.88E+01	2.83E+01
	Rank	1	8	6	4	3	7	5	9	2
F5	Best	6.00E+02	6.25E+02	6.19E+02	6.30E+02	6.03E+02	6.46E+02	6.34E+02	6.56E+02	6.03E+02
	Ave	6.00E+02	6.50E+02	6.36E+02	6.41E+02	6.09E+02	6.55E+02	6.51E+02	6.66E+02	6.08E+02
	Std	3.38E-01	1.30E+01	1.16E+01	7.04E+00	3.90E+00	6.14E+00	1.20E+01	4.99E+00	5.21E+00
	Rank	1	6	4	5	3	8	7	9	2
F6	Best	7.64E+02	8.92E+02	8.46E+02	9.55E+02	8.17E+02	1.00E+03	8.38E+02	1.21E+03	7.95E+02
	Ave	8.08E+02	1.19E+03	9.67E+02	1.06E+03	8.84E+02	1.11E+03	9.12E+02	1.30E+03	8.48E+02
	Std	1.95E+01	1.36E+02	7.78E+01	6.20E+01	5.64E+01	5.07E+01	6.45E+01	5.66E+01	3.06E+01
	Rank	1	8	5	6	3	7	4	9	2
F7	Best	8.41E+02	9.18E+02	9.15E+02	8.83E+02	8.60E+02	9.21E+02	8.95E+02	9.35E+02	8.53E+02
	Ave	8.76E+02	9.73E+02	9.96E+02	9.28E+02	8.94E+02	9.70E+02	9.60E+02	9.73E+02	8.99E+02
	Std	1.69E+01	2.45E+01	4.87E+01	2.55E+01	1.75E+01	2.36E+01	3.33E+01	2.30E+01	2.87E+01
	Rank	1	7	9	4	2	6	5	8	3
F8	Best	9.11E+02	2.92E+03	3.16E+03	1.68E+03	1.06E+03	3.93E+03	2.08E+03	5.50E+03	9.82E+02
	Ave	1.16E+03	6.87E+03	6.66E+03	3.51E+03	2.05E+03	6.93E+03	4.93E+03	8.05E+03	2.33E+03
	Std	2.85E+02	1.74E+03	2.20E+03	8.15E+02	8.54E+02	1.37E+03	1.57E+03	9.36E+02	1.54E+03
	Rank	1	7	6	4	2	8	5	9	3
F9	Best	3.20E+03	4.47E+03	4.20E+03	3.54E+03	3.22E+03	4.57E+03	3.92E+03	4.64E+03	3.23E+03
	Ave	5.26E+03	5.88E+03	5.76E+03	5.69E+03	4.87E+03	5.98E+03	5.02E+03	5.95E+03	4.46E+03
	Std	1.43E+03	8.09E+02	9.61E+02	1.03E+03	1.54E+03	6.84E+02	5.43E+02	6.54E+02	7.23E+02
	Rank	4	7	6	5	2	9	3	8	1
F10	Best	1.12E+03	1.23E+03	1.30E+03	1.15E+03	1.31E+03	1.87E+03	1.16E+03	1.24E+03	1.19E+03
	Ave	1.16E+03	1.45E+03	1.61E+03	1.25E+03	2.02E+03	3.02E+03	1.34E+03	1.38E+03	1.31E+03
	Std	2.82E+01	1.35E+02	2.44E+02	5.10E+01	9.04E+02	9.97E+02	7.82E+01	8.53E+01	6.01E+01
	Rank	1	6	7	2	8	9	4	5	3
F11	Best	5.49E+03	4.62E+05	1.21E+05	8.38E+04	1.76E+06	1.54E+07	1.79E+06	4.31E+06	1.21E+06
	Ave	5.08E+04	5.57E+06	3.48E+07	1.20E+06	5.81E+07	1.16E+08	2.17E+07	3.60E+07	1.51E+07
	Std	4.17E+04	3.23E+06	5.61E+07	1.11E+06	8.36E+07	9.67E+07	1.88E+07	2.58E+07	1.68E+07
	Rank	1	3	6	2	8	9	5	7	4
F12	Best	1.54E+03	2.37E+04	2.52E+04	2.01E+03	6.25E+04	1.52E+05	3.72E+04	2.84E+05	2.37E+04
	Ave	1.70E+04	1.43E+05	1.03E+07	1.23E+04	9.48E+06	6.14E+06	1.39E+05	8.29E+05	1.42E+05
	Std	2.17E+04	1.57E+05	2.05E+07	1.34E+04	3.34E+07	1.21E+07	8.72E+04	7.71E+05	1.69E+05
	Rank	2	5	9	1	8	7	3	6	4
F13	Best	1.49E+03	1.50E+04	5.06E+03	2.03E+03	7.97E+03	3.37E+04	5.17E+03	3.70E+04	1.32E+04
	Ave	1.56E+03	2.27E+05	1.57E+05	2.24E+04	6.01E+05	9.77E+05	3.90E+04	8.70E+05	6.93E+04
	Std	5.59E+01	2.33E+05	3.02E+05	2.32E+04	9.18E+05	9.60E+05	4.07E+04	7.99E+05	4.85E+04
	Rank	1	6	5	2	7	9	3	8	4
F14	Best	1.53E+03	4.56E+03	3.90E+03	1.62E+03	1.12E+04	2.22E+04	1.59E+04	2.47E+04	3.55E+03
	Ave	1.71E+03	2.07E+04	9.69E+04	6.79E+03	8.52E+05	1.55E+05	7.80E+04	1.14E+05	1.76E+04
	Std	1.58E+02	2.16E+04	1.57E+05	7.60E+03	1.57E+06	1.01E+05	6.77E+04	9.07E+04	1.25E+04
	Rank	1	4	6	2	9	8	5	7	3
F15	Best	1.85E+03	1.80E+03	2.32E+03	2.11E+03	1.88E+03	2.58E+03	2.07E+03	2.58E+03	2.07E+03
	Ave	2.34E+03	3.01E+03	3.20E+03	2.66E+03	2.58E+03	3.37E+03	2.71E+03	3.51E+03	2.64E+03
	Std	2.77E+02	4.19E+02	3.93E+02	2.72E+02	3.38E+02	5.04E+02	3.50E+02	4.54E+02	3.31E+02
	Rank	1	6	7	4	2	8	5	9	3
F16	Best	1.74E+03	1.95E+03	2.01E+03	1.86E+03	1.81E+03	1.90E+03	1.80E+03	2.04E+03	1.81E+03
	Ave	1.96E+03	2.22E+03	2.51E+03	2.28E+03	2.05E+03	2.48E+03	2.20E+03	2.62E+03	2.12E+03
	Std	1.42E+02	2.03E+02	2.73E+02	1.86E+02	1.44E+02	2.77E+02	2.00E+02	2.78E+02	1.63E+02
	Rank	1	5	8	6	2	7	4	9	3
F17	Best	2.01E+03	1.12E+05	1.01E+05	4.61E+04	1.04E+05	2.28E+05	9.39E+04	1.67E+05	6.26E+04
	Ave	1.16E+04	2.61E+06	2.82E+06	2.91E+05	2.08E+06	5.40E+06	1.24E+06	2.79E+06	1.02E+06
	Std	1.25E+04	2.49E+06	4.49E+06	3.08E+05	5.01E+06	4.38E+06	8.61E+05	2.95E+06	8.38E+05
	Rank	1	6	8	2	5	9	4	7	3
F18	Best	1.93E+03	2.40E+03	2.32E+03	2.18E+03	9.00E+03	1.00E+05	1.44E+05	4.34E+04	2.58E+03
	Ave	2.25E+03	1.26E+04	1.87E+06	8.07E+03	9.36E+05	3.04E+06	3.71E+06	1.05E+06	1.57E+04
	Std	9.39E+02	1.37E+04	2.84E+06	8.04E+03	1.63E+06	2.37E+06	2.15E+06	6.87E+05	1.38E+04
	Rank	1	3	7	2	5	8	9	6	4
F19	Best	2.07E+03	2.19E+03	2.34E+03	2.24E+03	2.15E+03	2.33E+03	2.22E+03	2.36E+03	2.11E+03
	Ave	2.32E+03	2.64E+03	2.68E+03	2.55E+03	2.43E+03	2.57E+03	2.57E+03	2.73E+03	2.43E+03
	Std	1.76E+02	2.33E+02	2.06E+02	1.70E+02	1.80E+02	1.52E+02	1.77E+02	1.91E+02	1.74E+02
	Rank	1	7	8	4	2	6	5	9	3
F20	Best	2.34E+03	2.38E+03	2.45E+03	2.38E+03	2.36E+03	2.43E+03	2.21E+03	2.48E+03	2.34E+03
	Ave	2.38E+03	2.45E+03	2.52E+03	2.44E+03	2.40E+03	2.50E+03	2.42E+03	2.57E+03	2.40E+03
	Std	2.32E+01	3.52E+01	3.90E+01	2.84E+01	3.23E+01	3.72E+01	4.71E+01	4.99E+01	2.99E+01
	Rank	1	6	8	5	2	7	4	9	3
F21	Best	2.30E+03	2.32E+03	2.33E+03	2.30E+03	2.40E+03	2.45E+03	2.30E+03	2.49E+03	2.31E+03
	Ave	3.58E+03	4.12E+03	5.50E+03	2.31E+03	5.26E+03	3.09E+03	3.82E+03	6.74E+03	4.34E+03
	Std	1.82E+03	2.70E+03	2.07E+03	4.54E+00	1.80E+03	1.10E+03	2.05E+03	1.73E+03	1.84E+03
	Rank	3	5	8	1	7	2	4	9	6
F22	Best	2.69E+03	2.74E+03	2.82E+03	2.75E+03	2.71E+03	2.87E+03	2.72E+03	3.02E+03	2.74E+03
	Ave	2.72E+03	2.84E+03	2.93E+03	2.91E+03	2.78E+03	3.00E+03	2.79E+03	3.22E+03	2.78E+03
	Std	1.46E+01	5.78E+01	7.47E+01	7.43E+01	4.72E+01	7.34E+01	4.53E+01	1.13E+02	2.80E+01
	Rank	1	5	7	6	3	8	4	9	2
F23	Best	2.87E+03	2.89E+03	2.96E+03	2.93E+03	2.87E+03	2.97E+03	2.88E+03	3.18E+03	2.86E+03
	Ave	2.90E+03	2.98E+03	3.09E+03	3.08E+03	2.95E+03	3.11E+03	2.93E+03	3.48E+03	2.93E+03
	Std	2.07E+01	4.79E+01	7.45E+01	1.02E+02	6.31E+01	6.20E+01	3.71E+01	1.46E+02	3.62E+01
	Rank	1	5	7	6	4	8	3	9	2
F24	Best	2.88E+03	2.91E+03	2.89E+03	2.89E+03	2.91E+03	2.96E+03	2.89E+03	2.93E+03	2.89E+03
	Ave	2.89E+03	2.95E+03	2.96E+03	2.92E+03	2.99E+03	3.03E+03	2.92E+03	2.98E+03	2.92E+03
	Std	1.20E+01	2.54E+01	5.43E+01	2.16E+01	6.54E+01	5.03E+01	1.97E+01	3.11E+01	2.38E+01
	Rank	1	5	6	2	8	9	4	7	3
F25	Best	2.80E+03	2.86E+03	3.78E+03	2.82E+03	3.64E+03	4.06E+03	2.80E+03	3.70E+03	2.85E+03
	Ave	3.96E+03	5.51E+03	6.42E+03	5.57E+03	4.82E+03	5.83E+03	4.56E+03	7.56E+03	4.84E+03
	Std	8.51E+02	1.42E+03	9.20E+02	1.18E+03	5.17E+02	1.40E+03	1.11E+03	1.39E+03	7.17E+02
	Rank	1	5	8	6	3	7	2	9	4
F26	Best	3.21E+03	3.21E+03	3.23E+03	3.23E+03	3.23E+03	3.28E+03	3.21E+03	3.30E+03	3.21E+03
	Ave	3.23E+03	3.27E+03	3.29E+03	3.30E+03	3.25E+03	3.37E+03	3.26E+03	3.48E+03	3.23E+03
	Std	1.17E+01	3.32E+01	5.04E+01	5.31E+01	1.98E+01	6.42E+01	4.05E+01	1.38E+02	1.32E+01
	Rank	1	5	6	7	3	8	4	9	2
F27	Best	3.10E+03	3.26E+03	3.30E+03	3.21E+03	3.29E+03	3.43E+03	3.22E+03	3.29E+03	3.23E+03
	Ave	3.18E+03	3.32E+03	3.58E+03	3.26E+03	3.42E+03	3.62E+03	3.27E+03	3.37E+03	3.27E+03
	Std	3.87E+01	2.27E+01	6.55E+02	2.47E+01	8.01E+01	1.39E+02	3.59E+01	6.32E+01	2.39E+01
	Rank	1	5	8	2	7	9	3	6	4
F28	Best	3.33E+03	3.61E+03	3.59E+03	3.53E+03	3.58E+03	4.07E+03	3.79E+03	3.94E+03	3.51E+03
	Ave	3.54E+03	4.12E+03	4.23E+03	4.19E+03	3.90E+03	4.72E+03	4.23E+03	4.66E+03	3.92E+03
	Std	1.26E+02	2.45E+02	3.02E+02	2.71E+02	1.74E+02	3.20E+02	2.50E+02	3.55E+02	2.40E+02
	Rank	1	4	7	5	2	9	6	8	3
F29	Best	5.56E+03	3.21E+04	1.63E+04	7.12E+03	8.46E+05	2.87E+06	3.98E+05	1.88E+06	8.16E+04
	Ave	8.12E+03	3.46E+05	1.82E+06	1.87E+04	9.96E+06	2.55E+07	7.06E+06	5.97E+06	6.00E+05
	Std	2.70E+03	2.85E+05	2.11E+06	8.08E+03	6.56E+06	2.27E+07	5.13E+06	3.36E+06	6.82E+05
	Rank	1	3	5	2	8	9	7	6	4

**Table A3:** CEC2017 experimental results (Dim = 50).

Function	Index	IMCOA	COA	DBO	TSO	GWO	AO	SSA	HHO	RIME
F1	Best	1.17E+02	1.10E+09	3.98E+08	2.97E+07	1.73E+09	6.71E+09	7.13E+04	5.14E+08	1.05E+07
	Ave	4.48E+03	5.68E+09	2.58E+09	1.39E+08	7.41E+09	1.13E+10	1.39E+05	1.62E+09	2.43E+07
	Std	5.79E+03	3.36E+09	4.36E+09	9.71E+07	2.96E+09	2.22E+09	4.20E+04	7.56E+08	7.48E+06
	Rank	1	7	6	4	8	9	2	5	3
F2	Best	9.44E+03	1.64E+05	1.83E+05	1.12E+05	9.46E+04	1.67E+05	8.38E+04	9.88E+04	7.87E+04
	Ave	2.76E+04	3.07E+05	2.86E+05	1.62E+05	1.35E+05	2.83E+05	1.66E+05	1.41E+05	1.49E+05
	Std	1.21E+04	8.07E+04	6.99E+04	2.58E+04	2.02E+04	5.80E+04	5.34E+04	1.91E+04	3.86E+04
	Rank	1	9	8	5	2	7	6	3	4
F3	Best	4.29E+02	6.66E+02	7.56E+02	5.66E+02	7.08E+02	1.60E+03	5.19E+02	7.75E+02	6.14E+02
	Ave	5.26E+02	1.10E+03	1.21E+03	7.08E+02	1.20E+03	2.42E+03	6.40E+02	1.17E+03	6.69E+02
	Std	5.54E+01	3.62E+02	7.16E+02	8.74E+01	4.21E+02	6.32E+02	5.05E+01	1.98E+02	4.27E+01
	Rank	1	5	8	4	7	9	2	6	3
F4	Best	6.26E+02	8.32E+02	7.91E+02	7.21E+02	6.65E+02	8.49E+02	7.08E+02	8.56E+02	6.45E+02
	Ave	6.73E+02	8.81E+02	9.61E+02	8.09E+02	7.35E+02	9.08E+02	8.51E+02	9.15E+02	7.07E+02
	Std	3.30E+01	2.21E+01	7.89E+01	4.66E+01	3.76E+01	3.90E+01	6.56E+01	3.05E+01	3.51E+01
	Rank	1	6	9	4	3	7	5	8	2
F5	Best	6.00E+02	6.38E+02	6.37E+02	6.43E+02	6.12E+02	6.55E+02	6.44E+02	6.65E+02	6.10E+02
	Ave	6.01E+02	6.66E+02	6.56E+02	6.56E+02	6.19E+02	6.69E+02	6.63E+02	6.77E+02	6.23E+02
	Std	6.24E-01	7.33E+00	1.08E+01	8.33E+00	3.72E+00	6.06E+00	9.48E+00	6.01E+00	6.94E+00
	Rank	1	7	5	4	2	8	6	9	3
F6	Best	8.50E+02	1.30E+03	9.86E+02	1.28E+03	9.62E+02	1.52E+03	1.01E+03	1.60E+03	9.53E+02
	Ave	9.00E+02	1.68E+03	1.22E+03	1.52E+03	1.10E+03	1.61E+03	1.21E+03	1.87E+03	1.05E+03
	Std	2.71E+01	1.42E+02	1.56E+02	1.30E+02	8.09E+01	6.89E+01	1.23E+02	9.60E+01	7.14E+01
	Rank	1	8	5	6	3	7	4	9	2
F7	Best	8.94E+02	1.14E+03	1.15E+03	9.77E+02	9.62E+02	1.15E+03	1.02E+03	1.13E+03	9.31E+02
	Ave	9.55E+02	1.21E+03	1.28E+03	1.10E+03	1.04E+03	1.23E+03	1.12E+03	1.21E+03	1.02E+03
	Std	3.52E+01	2.27E+01	8.28E+01	4.10E+01	4.19E+01	4.22E+01	7.69E+01	3.48E+01	4.76E+01
	Rank	1	7	9	4	3	8	5	6	2
F8	Best	1.05E+03	1.59E+04	9.85E+03	8.68E+03	3.21E+03	1.85E+04	9.50E+03	2.37E+04	2.99E+03
	Ave	2.37E+03	2.86E+04	2.20E+04	1.20E+04	9.17E+03	2.48E+04	1.46E+04	2.96E+04	7.19E+03
	Std	1.59E+03	7.00E+03	7.95E+03	2.38E+03	4.52E+03	3.94E+03	2.76E+03	3.41E+03	3.53E+03
	Rank	1	8	6	4	3	7	5	9	2
F9	Best	5.80E+03	1.09E+04	6.82E+03	7.55E+03	6.25E+03	8.22E+03	6.08E+03	8.61E+03	4.32E+03
	Ave	9.68E+03	1.31E+04	9.96E+03	9.36E+03	8.51E+03	1.01E+04	8.05E+03	9.88E+03	7.46E+03
	Std	2.83E+03	1.08E+03	1.99E+03	1.60E+03	2.44E+03	1.04E+03	1.18E+03	6.44E+02	1.01E+03
	Rank	5	9	7	4	3	8	2	6	1
F10	Best	1.16E+03	1.82E+03	1.90E+03	1.33E+03	3.24E+03	2.58E+03	1.51E+03	1.65E+03	1.45E+03
	Ave	1.24E+03	3.37E+03	3.56E+03	1.49E+03	7.10E+03	5.01E+03	1.81E+03	2.09E+03	1.67E+03
	Std	4.99E+01	1.65E+03	3.07E+03	9.07E+01	3.37E+03	1.20E+03	1.72E+02	2.60E+02	1.43E+02
	Rank	1	6	7	2	9	8	4	5	3
F11	Best	2.65E+05	3.42E+07	7.54E+07	6.12E+06	9.15E+07	6.76E+08	2.29E+07	7.92E+07	1.86E+07
	Ave	1.67E+06	1.18E+08	6.26E+08	1.58E+07	6.79E+08	2.31E+09	1.54E+08	4.13E+08	1.27E+08
	Std	1.19E+06	7.59E+07	5.16E+08	7.48E+06	8.26E+08	1.13E+09	1.28E+08	2.70E+08	7.79E+07
	Rank	1	3	7	2	8	9	5	6	4
F12	Best	1.50E+03	6.47E+04	4.78E+04	8.57E+03	1.72E+06	6.27E+06	5.36E+04	3.43E+06	9.01E+04
	Ave	9.17E+03	4.20E+05	1.06E+08	2.81E+04	1.72E+08	1.62E+08	2.07E+05	7.93E+06	3.93E+05
	Std	8.57E+03	2.65E+05	1.31E+08	1.80E+04	2.08E+08	1.23E+08	1.62E+05	4.24E+06	3.09E+05
	Rank	1	5	7	2	9	8	3	6	4
F13	Best	1.52E+03	1.44E+05	3.39E+04	1.34E+04	8.14E+04	9.24E+05	3.17E+04	3.43E+05	5.01E+04
	Ave	1.72E+03	1.38E+06	2.59E+06	1.51E+05	1.84E+06	6.05E+06	3.59E+05	3.01E+06	4.20E+05
	Std	1.22E+02	1.62E+06	3.43E+06	1.42E+05	2.38E+06	5.41E+06	3.07E+05	2.50E+06	3.18E+05
	Rank	1	5	7	2	6	9	3	8	4
F14	Best	1.66E+03	1.60E+04	4.04E+04	2.95E+03	3.87E+04	2.98E+05	1.63E+04	3.62E+05	2.84E+04
	Ave	8.10E+03	6.98E+04	5.62E+07	1.21E+04	1.86E+07	2.40E+06	6.88E+04	9.03E+05	9.48E+04
	Std	6.70E+03	4.35E+04	2.67E+08	8.46E+03	2.57E+07	2.32E+06	3.74E+04	4.27E+05	4.38E+04
	Rank	1	4	9	2	8	7	3	6	5
F15	Best	2.50E+03	2.56E+03	3.54E+03	2.93E+03	2.36E+03	3.14E+03	3.14E+03	3.73E+03	2.66E+03
	Ave	2.90E+03	3.74E+03	4.50E+03	3.64E+03	3.11E+03	4.62E+03	3.91E+03	4.70E+03	3.63E+03
	Std	3.03E+02	6.85E+02	4.64E+02	3.11E+02	4.20E+02	6.80E+02	5.79E+02	6.35E+02	5.38E+02
	Rank	1	5	7	4	2	8	6	9	3
F16	Best	2.12E+03	2.66E+03	3.11E+03	2.96E+03	2.37E+03	3.14E+03	2.55E+03	3.01E+03	2.64E+03
	Ave	2.66E+03	3.47E+03	3.98E+03	3.45E+03	2.94E+03	3.83E+03	3.46E+03	3.73E+03	3.25E+03
	Std	3.25E+02	4.35E+02	4.41E+02	2.81E+02	2.46E+02	4.01E+02	3.92E+02	3.97E+02	3.38E+02
	Rank	1	6	9	4	2	8	5	7	3
F17	Best	6.44E+03	2.51E+05	8.06E+05	1.36E+05	9.68E+05	7.06E+06	4.68E+05	5.14E+05	6.09E+05
	Ave	5.16E+04	5.77E+06	8.13E+06	1.18E+06	7.75E+06	2.36E+07	4.44E+06	7.82E+06	4.28E+06
	Std	2.65E+04	5.74E+06	8.70E+06	1.14E+06	7.79E+06	1.32E+07	3.65E+06	6.56E+06	3.21E+06
	Rank	1	5	8	2	6	9	4	7	3
F18	Best	1.96E+03	1.67E+04	1.48E+04	5.87E+03	1.51E+05	2.83E+05	1.30E+05	1.44E+05	3.68E+04
	Ave	1.53E+04	1.49E+05	4.24E+06	2.29E+04	5.63E+06	5.08E+06	7.08E+06	1.92E+06	3.55E+05
	Std	9.29E+03	1.35E+05	8.55E+06	9.89E+03	9.65E+06	4.66E+06	5.03E+06	1.63E+06	3.15E+05
	Rank	1	3	6	2	8	7	9	5	4
F19	Best	2.25E+03	2.92E+03	2.91E+03	2.73E+03	2.49E+03	2.94E+03	2.78E+03	2.94E+03	2.71E+03
	Ave	2.72E+03	3.66E+03	3.71E+03	3.23E+03	3.16E+03	3.39E+03	3.25E+03	3.58E+03	3.26E+03
	Std	2.70E+02	2.18E+02	3.68E+02	3.13E+02	3.99E+02	3.18E+02	2.81E+02	2.94E+02	3.19E+02
	Rank	1	8	9	3	2	6	4	7	5
F20	Best	2.40E+03	2.54E+03	2.57E+03	2.50E+03	2.46E+03	2.63E+03	2.50E+03	2.79E+03	2.46E+03
	Ave	2.45E+03	2.67E+03	2.80E+03	2.61E+03	2.52E+03	2.76E+03	2.62E+03	2.89E+03	2.52E+03
	Std	3.47E+01	8.15E+01	9.11E+01	7.69E+01	5.07E+01	6.26E+01	7.78E+01	7.41E+01	3.94E+01
	Rank	1	6	8	4	2	7	5	9	3
F21	Best	2.30E+03	2.59E+03	9.23E+03	8.34E+03	7.85E+03	1.03E+04	6.63E+03	1.02E+04	7.13E+03
	Ave	1.07E+04	1.42E+04	1.14E+04	1.13E+04	9.90E+03	1.23E+04	9.74E+03	1.19E+04	9.34E+03
	Std	3.99E+03	2.62E+03	1.26E+03	1.77E+03	2.54E+03	8.58E+02	9.93E+02	1.01E+03	9.26E+02
	Rank	4	9	6	5	3	8	2	7	1
F22	Best	2.79E+03	3.02E+03	3.14E+03	3.03E+03	2.90E+03	3.36E+03	2.94E+03	3.58E+03	2.88E+03
	Ave	2.87E+03	3.25E+03	3.39E+03	3.31E+03	3.01E+03	3.54E+03	3.05E+03	3.94E+03	2.99E+03
	Std	3.31E+01	1.27E+02	1.09E+02	1.22E+02	4.90E+01	1.12E+02	7.17E+01	1.58E+02	5.35E+01
	Rank	1	5	7	6	3	8	4	9	2
F23	Best	3.00E+03	3.16E+03	3.25E+03	3.23E+03	3.05E+03	3.28E+03	3.03E+03	3.84E+03	3.03E+03
	Ave	3.08E+03	3.34E+03	3.52E+03	3.62E+03	3.19E+03	3.57E+03	3.17E+03	4.25E+03	3.13E+03
	Std	4.70E+01	1.25E+02	1.37E+02	2.20E+02	9.89E+01	1.16E+02	7.62E+01	2.62E+02	6.55E+01
	Rank	1	5	6	8	4	7	3	9	2
F24	Best	3.00E+03	3.25E+03	2.99E+03	3.11E+03	3.29E+03	3.53E+03	3.05E+03	3.22E+03	3.08E+03
	Ave	3.05E+03	3.54E+03	3.32E+03	3.20E+03	3.64E+03	4.09E+03	3.12E+03	3.53E+03	3.14E+03
	Std	2.70E+01	2.22E+02	1.69E+02	5.76E+01	2.33E+02	2.66E+02	2.98E+01	1.25E+02	4.59E+01
	Rank	1	7	5	4	8	9	2	6	3
F25	Best	2.90E+03	7.24E+03	7.16E+03	4.13E+03	5.51E+03	6.61E+03	3.00E+03	6.00E+03	5.58E+03
	Ave	5.61E+03	1.14E+04	1.01E+04	1.01E+04	6.64E+03	1.02E+04	5.63E+03	1.15E+04	6.39E+03
	Std	1.09E+03	1.48E+03	1.19E+03	1.92E+03	5.93E+02	1.69E+03	2.31E+03	1.35E+03	5.14E+02
	Rank	1	8	6	5	4	7	2	9	3
F26	Best	3.27E+03	3.47E+03	3.45E+03	3.58E+03	3.47E+03	3.85E+03	3.47E+03	3.86E+03	3.43E+03
	Ave	3.37E+03	3.75E+03	3.75E+03	3.91E+03	3.65E+03	4.29E+03	3.62E+03	4.67E+03	3.56E+03
	Std	6.27E+01	1.43E+02	1.59E+02	2.62E+02	1.00E+02	2.07E+02	8.49E+01	4.28E+02	8.89E+01
	Rank	1	6	5	7	4	8	3	9	2
F27	Best	3.26E+03	3.52E+03	3.53E+03	3.44E+03	3.70E+03	4.65E+03	3.30E+03	3.73E+03	3.34E+03
	Ave	3.31E+03	4.03E+03	5.31E+03	3.56E+03	4.23E+03	5.49E+03	3.39E+03	4.29E+03	3.41E+03
	Std	3.06E+01	3.31E+02	1.88E+03	7.00E+01	2.98E+02	4.78E+02	6.03E+01	3.27E+02	5.70E+01
	Rank	1	5	8	4	6	9	2	7	3
F28	Best	3.52E+03	4.44E+03	4.82E+03	4.37E+03	4.01E+03	5.61E+03	4.54E+03	5.34E+03	4.14E+03
	Ave	3.96E+03	5.52E+03	5.75E+03	5.18E+03	4.64E+03	6.89E+03	5.61E+03	6.57E+03	4.93E+03
	Std	3.40E+02	5.98E+02	4.94E+02	3.95E+02	3.60E+02	6.23E+02	6.20E+02	6.78E+02	3.58E+02
	Rank	1	5	7	4	2	9	6	8	3
F29	Best	6.52E+05	7.58E+06	3.44E+06	8.32E+05	5.24E+07	6.90E+07	4.45E+07	3.69E+07	2.46E+07
	Ave	8.87E+05	2.07E+07	3.61E+07	2.03E+06	1.26E+08	1.66E+08	1.17E+08	9.27E+07	5.08E+07
	Std	1.39E+05	1.05E+07	3.82E+07	1.11E+06	4.72E+07	6.58E+07	4.20E+07	4.07E+07	1.65E+07
	Rank	1	3	4	2	8	9	7	6	5

**Table A4:** CEC2017 experimental results (Dim = 100).

Function	Index	IMCOA	COA	DBO	TSO	GWO	AO	SSA	HHO	RIME
F1	Best	1.30E+06	2.28E+10	1.05E+10	5.56E+09	2.75E+10	5.36E+10	1.63E+08	1.87E+10	3.42E+08
	Ave	3.51E+06	4.69E+10	5.46E+10	9.97E+09	4.60E+10	6.92E+10	5.02E+08	2.85E+10	5.72E+08
	Std	1.44E+06	9.07E+09	5.02E+10	3.16E+09	9.75E+09	7.42E+09	2.43E+08	5.27E+09	1.34E+08
	Rank	1	7	8	4	6	9	2	5	3
F2	Best	1.70E+05	4.87E+05	3.69E+05	3.28E+05	3.14E+05	3.27E+05	2.92E+05	3.00E+05	5.46E+05
	Ave	2.51E+05	6.88E+05	7.45E+05	4.13E+05	4.63E+05	3.53E+05	4.49E+05	3.30E+05	6.40E+05
	Std	4.13E+04	1.15E+05	2.50E+05	6.49E+04	7.37E+04	9.80E+03	1.45E+05	1.82E+04	5.84E+04
	Rank	1	8	9	4	6	3	5	2	7
F3	Best	6.56E+02	3.05E+03	2.51E+03	1.70E+03	2.48E+03	9.22E+03	9.33E+02	4.25E+03	8.59E+02
	Ave	7.32E+02	5.48E+03	1.45E+04	2.13E+03	4.11E+03	1.27E+04	1.12E+03	5.74E+03	1.10E+03
	Std	5.15E+01	1.05E+03	1.04E+04	3.10E+02	7.63E+02	1.98E+03	1.03E+02	1.23E+03	1.27E+02
	Rank	1	6	9	4	5	8	3	7	2
F4	Best	8.23E+02	1.39E+03	1.31E+03	1.14E+03	1.05E+03	1.52E+03	1.13E+03	1.54E+03	1.02E+03
	Ave	9.09E+02	1.48E+03	1.74E+03	1.37E+03	1.19E+03	1.64E+03	1.39E+03	1.63E+03	1.18E+03
	Std	5.62E+01	4.96E+01	2.02E+02	7.98E+01	1.17E+02	6.38E+01	1.17E+02	5.79E+01	8.95E+01
	Rank	1	6	9	4	3	8	5	7	2
F5	Best	6.02E+02	6.67E+02	6.53E+02	6.61E+02	6.32E+02	6.73E+02	6.63E+02	6.83E+02	6.32E+02
	Ave	6.04E+02	6.71E+02	6.76E+02	6.70E+02	6.40E+02	6.86E+02	6.71E+02	6.90E+02	6.44E+02
	Std	1.66E+00	2.44E+00	1.32E+01	4.68E+00	3.73E+00	3.98E+00	5.74E+00	3.79E+00	7.18E+00
	Rank	1	5	7	4	2	8	6	9	3
F6	Best	1.19E+03	2.78E+03	2.25E+03	2.69E+03	1.76E+03	3.04E+03	1.85E+03	3.46E+03	1.67E+03
	Ave	1.29E+03	3.30E+03	2.59E+03	3.06E+03	1.99E+03	3.37E+03	2.35E+03	3.76E+03	1.97E+03
	Std	7.92E+01	1.68E+02	2.96E+02	1.83E+02	9.47E+01	1.34E+02	2.55E+02	1.14E+02	1.51E+02
	Rank	1	7	5	6	3	8	4	9	2
F7	Best	1.11E+03	1.83E+03	1.62E+03	1.58E+03	1.37E+03	1.77E+03	1.57E+03	1.92E+03	1.38E+03
	Ave	1.21E+03	1.98E+03	2.06E+03	1.77E+03	1.50E+03	2.07E+03	1.76E+03	2.08E+03	1.50E+03
	Std	6.46E+01	5.36E+01	2.03E+02	8.93E+01	1.02E+02	8.86E+01	1.25E+02	7.68E+01	7.55E+01
	Rank	1	6	7	5	3	8	4	9	2
F8	Best	2.67E+03	3.25E+04	3.06E+04	2.64E+04	1.95E+04	5.28E+04	2.49E+04	5.27E+04	1.97E+04
	Ave	2.42E+04	4.98E+04	6.49E+04	3.21E+04	3.61E+04	6.18E+04	3.43E+04	6.49E+04	3.73E+04
	Std	1.36E+04	1.32E+04	1.77E+04	2.89E+03	1.01E+04	4.91E+03	3.53E+03	5.54E+03	1.20E+04
	Rank	1	6	8	2	4	7	3	9	5
F9	Best	1.41E+04	1.86E+04	1.68E+04	1.74E+04	1.48E+04	2.10E+04	1.38E+04	2.02E+04	1.55E+04
	Ave	2.25E+04	2.32E+04	2.14E+04	2.26E+04	2.03E+04	2.46E+04	1.67E+04	2.33E+04	1.81E+04
	Std	5.53E+03	3.05E+03	4.12E+03	3.81E+03	6.30E+03	1.88E+03	1.41E+03	1.77E+03	1.28E+03
	Rank	5	7	4	6	3	9	1	8	2
F10	Best	1.84E+03	1.34E+05	1.09E+05	3.46E+04	5.20E+04	1.48E+05	3.83E+04	4.24E+04	1.07E+04
	Ave	2.09E+03	2.60E+05	2.06E+05	6.71E+04	7.29E+04	2.92E+05	6.19E+04	9.49E+04	2.08E+04
	Std	1.97E+02	5.97E+04	5.52E+04	1.77E+04	1.25E+04	7.91E+04	1.57E+04	2.70E+04	6.63E+03
	Rank	1	8	7	4	5	9	3	6	2
F11	Best	5.98E+06	1.08E+09	1.59E+09	2.30E+08	3.62E+09	1.18E+10	1.73E+08	2.34E+09	1.87E+08
	Ave	2.03E+07	3.67E+09	4.36E+09	5.47E+08	9.22E+09	1.98E+10	9.10E+08	4.94E+09	9.88E+08
	Std	8.70E+06	2.42E+09	1.78E+09	2.37E+08	5.22E+09	5.28E+09	4.30E+08	1.54E+09	4.63E+08
	Rank	1	5	6	2	8	9	3	7	4
F12	Best	1.87E+03	6.54E+05	2.98E+06	6.33E+04	1.31E+08	4.24E+08	6.21E+04	2.42E+07	6.37E+05
	Ave	7.36E+03	6.66E+07	1.49E+08	2.62E+05	1.31E+09	1.16E+09	1.04E+05	5.28E+07	1.35E+06
	Std	6.24E+03	2.25E+08	1.24E+08	3.63E+05	1.55E+09	4.51E+08	2.88E+04	3.96E+07	1.03E+06
	Rank	1	6	7	3	9	8	2	5	4
F13	Best	1.32E+04	1.08E+06	2.28E+06	3.54E+05	1.61E+06	6.27E+06	1.11E+06	4.70E+06	1.85E+06
	Ave	9.46E+04	6.74E+06	1.27E+07	1.07E+06	7.16E+06	1.76E+07	4.28E+06	7.77E+06	7.20E+06
	Std	1.02E+05	3.31E+06	9.35E+06	5.97E+05	4.15E+06	6.31E+06	1.72E+06	1.85E+06	2.87E+06
	Rank	1	4	8	2	5	9	3	7	6
F14	Best	1.77E+03	9.14E+04	1.36E+05	1.02E+04	7.00E+05	1.43E+07	2.61E+04	2.23E+06	1.49E+05
	Ave	4.31E+03	1.50E+06	2.32E+07	2.47E+04	2.23E+08	1.49E+08	8.03E+04	5.53E+06	3.32E+05
	Std	4.98E+03	2.64E+06	3.74E+07	1.04E+04	4.55E+08	1.11E+08	3.02E+04	1.76E+06	2.88E+05
	Rank	1	5	7	2	9	8	3	6	4
F15	Best	3.85E+03	6.37E+03	6.27E+03	4.89E+03	5.04E+03	8.57E+03	4.76E+03	7.64E+03	5.61E+03
	Ave	4.88E+03	8.04E+03	8.39E+03	6.83E+03	6.57E+03	1.12E+04	7.24E+03	9.32E+03	6.92E+03
	Std	5.68E+02	1.48E+03	9.24E+02	8.16E+02	8.08E+02	1.40E+03	8.66E+02	9.94E+02	6.96E+02
	Rank	1	6	7	3	2	9	5	8	4
F16	Best	3.36E+03	4.86E+03	6.49E+03	5.38E+03	3.88E+03	7.34E+03	4.69E+03	5.44E+03	4.46E+03
	Ave	4.30E+03	6.31E+03	8.63E+03	6.21E+03	5.46E+03	1.19E+04	5.68E+03	7.04E+03	5.55E+03
	Std	5.13E+02	6.73E+02	1.26E+03	5.45E+02	1.07E+03	3.65E+03	5.97E+02	8.38E+02	5.77E+02
	Rank	1	6	8	5	2	9	4	7	3
F17	Best	1.04E+05	2.07E+06	4.12E+06	5.43E+05	7.68E+05	4.20E+06	1.69E+06	3.48E+06	3.03E+06
	Ave	3.93E+05	7.65E+06	1.71E+07	2.40E+06	7.42E+06	1.64E+07	6.93E+06	8.98E+06	9.31E+06
	Std	3.53E+05	4.31E+06	1.10E+07	1.55E+06	3.98E+06	8.48E+06	4.43E+06	4.26E+06	4.32E+06
	Rank	1	5	9	2	4	8	3	6	7
F18	Best	2.00E+03	1.38E+06	5.07E+06	9.12E+03	1.24E+07	3.35E+07	4.38E+06	8.16E+06	2.40E+06
	Ave	5.57E+03	6.44E+06	5.10E+07	1.52E+05	1.69E+08	1.30E+08	2.24E+07	2.21E+07	1.39E+07
	Std	6.19E+03	3.37E+06	6.38E+07	1.37E+05	2.13E+08	1.15E+08	1.27E+07	1.16E+07	7.12E+06
	Rank	1	3	7	2	9	8	6	5	4
F19	Best	3.60E+03	5.54E+03	4.93E+03	4.35E+03	4.04E+03	4.76E+03	4.66E+03	4.84E+03	4.46E+03
	Ave	5.43E+03	6.99E+03	6.59E+03	5.63E+03	5.67E+03	6.10E+03	5.53E+03	5.93E+03	5.54E+03
	Std	1.15E+03	5.57E+02	8.16E+02	7.30E+02	1.32E+03	5.91E+02	5.32E+02	4.79E+02	4.90E+02
	Rank	1	9	8	4	5	7	2	6	3
F20	Best	2.57E+03	3.40E+03	3.53E+03	3.02E+03	2.92E+03	3.64E+03	2.98E+03	3.97E+03	2.87E+03
	Ave	2.67E+03	3.60E+03	3.83E+03	3.36E+03	3.06E+03	4.11E+03	3.24E+03	4.24E+03	3.03E+03
	Std	5.04E+01	1.74E+02	1.68E+02	1.33E+02	1.24E+02	3.04E+02	1.44E+02	1.72E+02	8.64E+01
	Rank	1	6	7	5	3	8	4	9	2
F21	Best	2.32E+03	2.37E+04	1.91E+04	2.01E+04	1.76E+04	2.41E+04	2.44E+03	2.32E+04	1.76E+04
	Ave	2.74E+04	2.82E+04	2.39E+04	2.57E+04	2.08E+04	2.70E+04	1.98E+04	2.67E+04	2.11E+04
	Std	6.54E+03	2.23E+03	4.33E+03	3.38E+03	2.93E+03	1.41E+03	3.73E+03	1.97E+03	1.94E+03
	Rank	8	9	4	5	2	7	1	6	3
F22	Best	3.02E+03	3.85E+03	4.03E+03	3.98E+03	3.49E+03	4.47E+03	3.44E+03	4.57E+03	3.37E+03
	Ave	3.09E+03	4.11E+03	4.43E+03	4.60E+03	3.62E+03	4.88E+03	3.66E+03	5.38E+03	3.57E+03
	Std	3.69E+01	1.72E+02	1.91E+02	3.24E+02	8.91E+01	2.55E+02	1.61E+02	3.20E+02	1.09E+02
	Rank	1	5	6	7	3	8	4	9	2
F23	Best	3.52E+03	4.51E+03	4.81E+03	5.35E+03	3.91E+03	5.45E+03	4.06E+03	6.57E+03	3.85E+03
	Ave	3.61E+03	5.07E+03	5.50E+03	6.22E+03	4.23E+03	6.26E+03	4.26E+03	7.48E+03	4.08E+03
	Std	4.15E+01	3.33E+02	3.51E+02	7.89E+02	1.45E+02	3.72E+02	1.48E+02	5.59E+02	1.45E+02
	Rank	1	5	6	7	3	8	4	9	2
F24	Best	3.27E+03	5.05E+03	4.69E+03	3.90E+03	4.94E+03	6.57E+03	3.64E+03	5.12E+03	3.55E+03
	Ave	3.34E+03	6.49E+03	9.84E+03	4.39E+03	6.19E+03	8.06E+03	3.95E+03	5.64E+03	3.81E+03
	Std	5.22E+01	7.68E+02	6.20E+03	2.12E+02	7.14E+02	7.63E+02	1.39E+02	2.84E+02	9.10E+01
	Rank	1	7	9	4	6	8	3	5	2
F25	Best	2.96E+03	2.36E+04	1.93E+04	2.08E+04	1.43E+04	2.54E+04	7.11E+03	1.78E+04	1.11E+04
	Ave	8.72E+03	3.08E+04	2.57E+04	2.71E+04	1.58E+04	3.12E+04	1.63E+04	2.95E+04	1.42E+04
	Std	2.26E+03	2.84E+03	3.50E+03	2.86E+03	1.05E+03	2.40E+03	2.86E+03	2.94E+03	1.33E+03
	Rank	1	8	5	6	3	9	4	7	2
F26	Best	3.43E+03	3.92E+03	3.75E+03	3.91E+03	3.92E+03	5.12E+03	3.73E+03	4.32E+03	3.70E+03
	Ave	3.48E+03	4.37E+03	4.28E+03	4.39E+03	4.17E+03	6.30E+03	4.04E+03	5.69E+03	3.93E+03
	Std	2.99E+01	2.99E+02	3.91E+02	3.68E+02	1.49E+02	5.94E+02	1.54E+02	6.03E+02	1.10E+02
	Rank	1	6	5	7	4	9	3	8	2
F27	Best	3.38E+03	6.23E+03	5.63E+03	4.40E+03	6.81E+03	1.02E+04	3.81E+03	6.34E+03	3.77E+03
	Ave	3.46E+03	8.69E+03	1.72E+04	5.05E+03	8.43E+03	1.19E+04	4.22E+03	7.55E+03	4.03E+03
	Std	3.98E+01	1.29E+03	5.94E+03	4.46E+02	1.17E+03	1.20E+03	3.71E+02	6.93E+02	1.50E+02
	Rank	1	7	9	4	6	8	3	5	2
F28	Best	5.18E+03	8.59E+03	8.01E+03	7.09E+03	7.65E+03	1.13E+04	8.39E+03	1.03E+04	6.95E+03
	Ave	6.05E+03	1.03E+04	1.08E+04	9.26E+03	8.61E+03	1.53E+04	1.01E+04	1.18E+04	8.83E+03
	Std	5.93E+02	1.17E+03	1.69E+03	8.57E+02	5.28E+02	2.02E+03	9.58E+02	1.04E+03	8.85E+02
	Rank	1	6	7	4	2	9	5	8	3
F29	Best	9.03E+03	1.84E+07	1.34E+07	3.30E+06	5.53E+07	6.12E+08	4.01E+07	1.12E+08	4.68E+07
	Ave	2.39E+04	7.92E+07	1.33E+08	9.41E+06	7.26E+08	1.62E+09	2.26E+08	2.75E+08	1.42E+08
	Std	1.29E+04	3.98E+07	1.08E+08	6.36E+06	6.45E+08	6.04E+08	9.80E+07	1.27E+08	7.20E+07
	Rank	1	3	4	2	8	9	6	7	5

**Table A5:** CEC2022 test functions.

Type	ID	CEC2022 Function Name	Range	Dimension	*f*_min_
Unimodal function	F1	Shifted and full Rotated Zakharov Function	[−100, 100]	10/20	300
Basic Functions	F2	Shifted and full Rotated Rosenbrock’s Function	[−100, 100]	10/20	400
	F3	Shifted and full Expanded Schaffer’s f6 Function	[−100, 100]	10/20	600
	F4	Shifted and full Rotated Non-Continuous Rastrigin’s Function	[−100, 100]	10/20	800
	F5	Shifted and full Rotated Levy Function	[−100, 100]	10/20	900
Hybrid functions	F6	Hybrid Function 1 (N = 3)	[−100, 100]	10/20	1800
	F7	Hybrid Function 2 (N = 6)	[−100, 100]	10/20	2000
	F8	Hybrid Function 3 (N = 5)	[−100, 100]	10/20	2200
Composition functions	F9	Composition Function 1 (N = 5)	[−100, 100]	10/20	2300
	F10	Composition Function 2 (N = 4)	[−100, 100]	10/20	2400
	F11	Composition Function 3 (N = 5)	[−100, 100]	10/20	2600
	F12	Composition Function 5 (N = 6)	[−100, 100]	10/20	2700

**Table A6:** CEC2022 experimental results (Dim = 10).

Function	Index	IMCOA	COA	DBO	TSO	GWO	AO	SSA	HHO	RIME
F1	Best	3.00E+02	3.70E+02	3.00E+02	3.00E+02	3.60E+02	1.16E+03	3.00E+02	3.03E+02	3.00E+02
	Ave	3.00E+02	2.05E+03	4.58E+02	3.00E+02	1.97E+03	2.94E+03	3.00E+02	3.93E+02	3.00E+02
	Std	3.84E-10	1.72E+03	3.22E+02	5.60E-02	1.80E+03	1.88E+03	7.39E-04	9.56E+01	2.26E-01
	Rank	1	8	6	3	7	9	2	5	4
F2	Best	4.00E+02	4.00E+02	4.00E+02	4.00E+02	4.04E+02	4.01E+02	4.00E+02	4.00E+02	4.00E+02
	Ave	4.04E+02	4.23E+02	4.22E+02	4.09E+02	4.19E+02	4.26E+02	4.06E+02	4.40E+02	4.09E+02
	Std	3.50E+00	3.11E+01	2.98E+01	1.66E+01	1.66E+01	1.93E+01	2.82E+00	4.63E+01	1.24E+01
	Rank	1	7	6	3	5	8	2	9	4
F3	Best	6.00E+02	6.00E+02	6.00E+02	6.00E+02	6.00E+02	6.06E+02	6.01E+02	6.11E+02	6.00E+02
	Ave	6.00E+02	6.06E+02	6.04E+02	6.08E+02	6.02E+02	6.18E+02	6.13E+02	6.38E+02	6.00E+02
	Std	4.03E-02	1.03E+01	3.64E+00	6.23E+00	2.31E+00	5.94E+00	1.11E+01	1.39E+01	8.44E-02
	Rank	1	5	4	6	3	8	7	9	2
F4	Best	8.05E+02	8.12E+02	8.11E+02	8.06E+02	8.03E+02	8.09E+02	8.08E+02	8.08E+02	8.11E+02
	Ave	8.13E+02	8.27E+02	8.29E+02	8.18E+02	8.13E+02	8.20E+02	8.23E+02	8.25E+02	8.25E+02
	Std	6.58E+00	7.67E+00	1.22E+01	6.59E+00	6.79E+00	6.91E+00	1.17E+01	8.80E+00	1.03E+01
	Rank	1	8	9	3	2	4	5	7	6
F5	Best	9.00E+02	9.00E+02	9.00E+02	9.03E+02	9.00E+02	9.03E+02	9.00E+02	9.86E+02	9.00E+02
	Ave	9.02E+02	1.00E+03	9.45E+02	9.70E+02	9.05E+02	1.01E+03	9.61E+02	1.32E+03	9.00E+02
	Std	4.07E+00	2.09E+02	7.52E+01	5.89E+01	1.27E+01	6.06E+01	1.45E+02	1.73E+02	4.60E-01
	Rank	2	7	4	6	3	8	5	9	1
F6	Best	1.80E+03	1.98E+03	1.83E+03	1.83E+03	2.85E+03	3.10E+03	1.90E+03	2.07E+03	1.92E+03
	Ave	1.82E+03	4.35E+03	5.14E+03	2.86E+03	6.70E+03	1.91E+04	4.33E+03	4.40E+03	4.48E+03
	Std	1.51E+01	1.71E+03	2.43E+03	1.17E+03	2.17E+03	1.61E+04	2.15E+03	1.64E+03	1.88E+03
	Rank	1	4	7	2	8	9	3	5	6
F7	Best	2.00E+03	2.00E+03	2.02E+03	2.00E+03	2.01E+03	2.02E+03	2.00E+03	2.03E+03	2.00E+03
	Ave	2.02E+03	2.02E+03	2.03E+03	2.02E+03	2.03E+03	2.05E+03	2.04E+03	2.07E+03	2.02E+03
	Std	9.15E+00	7.39E+00	1.15E+01	1.35E+01	1.06E+01	1.53E+01	1.58E+01	2.74E+01	2.47E+01
	Rank	1	2	5	4	6	8	7	9	3
F8	Best	2.20E+03	2.21E+03	2.22E+03	2.20E+03	2.22E+03	2.22E+03	2.21E+03	2.22E+03	2.20E+03
	Ave	2.22E+03	2.22E+03	2.23E+03	2.22E+03	2.23E+03	2.23E+03	2.23E+03	2.24E+03	2.22E+03
	Std	8.39E+00	6.38E+00	4.00E+00	5.11E+00	2.82E+00	3.87E+00	6.82E+00	1.35E+01	7.34E+00
	Rank	1	4	5	3	6	8	7	9	2
F9	Best	2.53E+03	2.53E+03	2.53E+03	2.53E+03	2.53E+03	2.55E+03	2.53E+03	2.53E+03	2.53E+03
	Ave	2.53E+03	2.54E+03	2.54E+03	2.53E+03	2.56E+03	2.59E+03	2.54E+03	2.58E+03	2.53E+03
	Std	2.68E+01	4.48E+01	3.48E+01	2.68E+01	3.23E+01	2.81E+01	2.33E+01	4.13E+01	2.68E+01
	Rank	1	5	6	1	7	9	4	8	3
F10	Best	2.50E+03	2.50E+03	2.50E+03	2.50E+03	2.50E+03	2.50E+03	2.50E+03	2.50E+03	2.50E+03
	Ave	2.55E+03	2.55E+03	2.53E+03	2.52E+03	2.54E+03	2.57E+03	2.50E+03	2.58E+03	2.54E+03
	Std	5.74E+01	6.13E+01	5.64E+01	4.49E+01	5.61E+01	5.96E+01	2.24E+01	9.98E+01	5.42E+01
	Rank	6	7	3	2	5	8	1	9	4
F11	Best	2.60E+03	2.60E+03	2.60E+03	2.60E+03	2.75E+03	2.61E+03	2.60E+03	2.61E+03	2.60E+03
	Ave	2.85E+03	2.79E+03	2.86E+03	2.71E+03	2.94E+03	2.74E+03	2.94E+03	2.83E+03	2.90E+03
	Std	1.07E+02	1.42E+02	3.66E+02	1.40E+02	8.62E+01	1.04E+02	3.63E+02	1.16E+02	6.45E+01
	Rank	5	3	6	1	9	2	8	4	7
F12	Best	2.86E+03	2.86E+03	2.86E+03	2.86E+03	2.86E+03	2.86E+03	2.86E+03	2.86E+03	2.86E+03
	Ave	2.86E+03	2.87E+03	2.87E+03	2.87E+03	2.87E+03	2.87E+03	2.86E+03	2.90E+03	2.87E+03
	Std	1.68E+00	3.86E+00	9.12E+00	1.95E+00	6.65E+00	2.75E+00	1.67E+00	4.14E+01	2.18E+00
	Rank	2	6	8	4	5	7	1	9	3

**Table A7:** CEC2022 experimental results (Dim = 20).

Function	Index	IMCOA	COA	DBO	TSO	GWO	AO	SSA	HHO	RIME
F1	Best	3.00E+02	1.91E+04	9.51E+03	2.35E+03	2.31E+03	2.35E+04	3.05E+02	4.06E+03	3.87E+02
	Ave	3.00E+02	4.04E+04	2.56E+04	6.25E+03	1.17E+04	5.40E+04	1.03E+03	1.30E+04	6.03E+02
	Std	1.37E-05	1.46E+04	1.01E+04	2.42E+03	5.62E+03	1.65E+04	6.34E+02	4.57E+03	2.21E+02
	Rank	1	8	7	4	5	9	3	6	2
F2	Best	4.00E+02	4.45E+02	4.07E+02	4.00E+02	4.54E+02	4.68E+02	4.34E+02	4.59E+02	4.06E+02
	Ave	4.39E+02	4.74E+02	4.82E+02	4.58E+02	5.00E+02	5.42E+02	4.59E+02	5.31E+02	4.52E+02
	Std	2.20E+01	2.52E+01	6.30E+01	2.01E+01	3.49E+01	4.40E+01	1.75E+01	4.65E+01	1.61E+01
	Rank	1	5	6	3	7	9	4	8	2
F3	Best	6.00E+02	6.01E+02	6.07E+02	6.16E+02	6.01E+02	6.29E+02	6.11E+02	6.45E+02	6.01E+02
	Ave	6.00E+02	6.30E+02	6.22E+02	6.28E+02	6.04E+02	6.43E+02	6.34E+02	6.62E+02	6.02E+02
	Std	1.22E-01	1.93E+01	9.87E+00	8.06E+00	2.46E+00	8.00E+00	1.63E+01	8.27E+00	1.40E+00
	Rank	1	6	4	5	3	8	7	9	2
F4	Best	8.21E+02	8.60E+02	8.40E+02	8.30E+02	8.25E+02	8.55E+02	8.41E+02	8.54E+02	8.24E+02
	Ave	8.46E+02	8.87E+02	9.06E+02	8.54E+02	8.59E+02	8.74E+02	8.83E+02	8.88E+02	8.52E+02
	Std	1.09E+01	1.01E+01	2.87E+01	1.41E+01	3.10E+01	1.37E+01	2.51E+01	1.43E+01	1.57E+01
	Rank	1	7	9	3	4	5	6	8	2
F5	Best	9.00E+02	1.37E+03	9.65E+02	1.11E+03	9.05E+02	1.49E+03	9.67E+02	2.09E+03	9.01E+02
	Ave	1.01E+03	2.55E+03	2.07E+03	1.71E+03	1.11E+03	2.19E+03	1.90E+03	2.84E+03	1.05E+03
	Std	1.39E+02	6.00E+02	7.46E+02	3.65E+02	1.49E+02	4.06E+02	6.81E+02	3.39E+02	2.21E+02
	Rank	1	8	6	4	3	7	5	9	2
F6	Best	1.85E+03	2.05E+03	2.00E+03	1.88E+03	4.26E+03	1.95E+04	2.18E+03	2.20E+04	2.34E+03
	Ave	2.01E+03	6.83E+03	3.46E+05	6.75E+03	9.35E+05	2.47E+05	9.77E+03	1.65E+05	1.10E+04
	Std	2.97E+02	6.73E+03	5.88E+05	6.31E+03	2.84E+06	2.11E+05	7.23E+03	1.03E+05	6.78E+03
	Rank	1	3	8	2	9	7	4	6	5
F7	Best	2.02E+03	2.03E+03	2.04E+03	2.06E+03	2.03E+03	2.08E+03	2.03E+03	2.13E+03	2.03E+03
	Ave	2.05E+03	2.10E+03	2.12E+03	2.10E+03	2.08E+03	2.12E+03	2.10E+03	2.20E+03	2.06E+03
	Std	1.91E+01	5.20E+01	5.50E+01	2.54E+01	4.81E+01	2.11E+01	4.35E+01	6.63E+01	3.30E+01
	Rank	1	6	8	4	3	7	5	9	2
F8	Best	2.22E+03	2.23E+03	2.23E+03	2.22E+03	2.23E+03	2.23E+03	2.22E+03	2.23E+03	2.22E+03
	Ave	2.25E+03	2.28E+03	2.29E+03	2.23E+03	2.26E+03	2.25E+03	2.29E+03	2.29E+03	2.24E+03
	Std	5.26E+01	7.24E+01	6.56E+01	9.05E+00	5.30E+01	3.69E+01	6.69E+01	8.96E+01	3.91E+01
	Rank	3	6	9	1	5	4	8	7	2
F9	Best	2.48E+03	2.48E+03	2.48E+03	2.48E+03	2.48E+03	2.52E+03	2.48E+03	2.49E+03	2.48E+03
	Ave	2.48E+03	2.48E+03	2.52E+03	2.48E+03	2.51E+03	2.56E+03	2.52E+03	2.52E+03	2.48E+03
	Std	5.19E-08	1.99E-01	4.20E+01	8.95E-03	2.73E+01	3.12E+01	5.34E+01	2.53E+01	4.02E-01
	Rank	1	3	6	2	5	9	8	7	4
F10	Best	2.50E+03	2.50E+03	2.50E+03	2.50E+03	2.50E+03	2.50E+03	2.50E+03	2.50E+03	2.50E+03
	Ave	2.71E+03	3.59E+03	3.03E+03	2.65E+03	3.81E+03	3.18E+03	3.79E+03	3.90E+03	2.65E+03
	Std	2.81E+02	1.16E+03	9.68E+02	4.45E+02	8.70E+02	1.05E+03	1.23E+03	8.20E+02	1.71E+02
	Rank	3	6	4	1	8	5	7	9	2
F11	Best	2.90E+03	2.91E+03	2.60E+03	2.90E+03	3.10E+03	3.12E+03	2.61E+03	2.97E+03	2.96E+03
	Ave	2.90E+03	2.99E+03	2.90E+03	2.92E+03	3.53E+03	3.64E+03	2.90E+03	3.31E+03	3.00E+03
	Std	7.61E-04	1.18E+02	7.98E+01	7.20E+01	3.49E+02	4.89E+02	5.58E+01	4.89E+02	2.50E+01
	Rank	1	5	2	4	8	9	3	7	6
F12	Best	2.94E+03	2.94E+03	2.95E+03	2.95E+03	2.95E+03	2.97E+03	2.94E+03	2.97E+03	2.94E+03
	Ave	2.96E+03	2.98E+03	3.01E+03	3.03E+03	2.97E+03	3.03E+03	2.97E+03	3.14E+03	2.96E+03
	Std	1.98E+01	3.70E+01	6.90E+01	7.86E+01	1.34E+01	3.65E+01	2.48E+01	1.46E+02	1.45E+01
	Rank	2	5	6	8	3	7	4	9	1
